# Stability and bifurcation analysis of a 2DOF dynamical system with piezoelectric device and feedback control

**DOI:** 10.1038/s41598-024-75342-z

**Published:** 2024-11-02

**Authors:** Taher A. Bahnasy, T. S. Amer, M. K. Abohamer, H. F. Abosheiaha, A. S. Elameer, A. Almahalawy

**Affiliations:** 1 Department of Physics and Engineering Mathematics, Faculty of Engineering, Tanta University, Tanta, 31734 Egypt; 2 Mathematics Department, Faculty of Science, Tanta University, Tanta, 31527 Egypt; 3 Basic Science Center, Misr University for Science and Technology (MUST), 6 of October, Egypt

**Keywords:** Dynamical system, Spring pendulum, Energy harvesting, Negative velocity feedback controller, Perturbation techniques, Piezoelectric device, Stability, Lyapunov exponent, Bifurcation analysis, Poincaré maps., Engineering, Mathematics and computing

## Abstract

This study aims to demonstrate the behaviors of a two degree-of-freedom (DOF) dynamical system consisting of attached mass to a nonlinear damped harmonic spring pendulum with a piezoelectric device. Such a system is influenced by a parametric excitation force on the direction of the spring’s elongation and an operating moment at the supported point. A negative-velocity-feedback (NVF) controller is inserted into the main system to reduce the undesired vibrations that affect the system’s efficiency, especially at the resonance state. The equations of motion (EOM) are derived by using Lagrangian equations. Through the use of the multiple-scales-strategy (MSS), approximate solutions (AS) are investigated up to the third order. The accuracy of the AS is verified by comparing them to the obtained numerical solutions (NS) through the fourth-order Runge-Kutta Method (RK-4). The study delves into resonance cases and solvability conditions to provide the modulation equations (ME). Graphical representations showing the time histories of the obtained solutions and frequency responses are presented utilizing Wolfram Mathematica 13.2 in addition to MATLAB software. Additionally, discusses the bifurcation diagrams, Poincaré maps, and Lyapunov exponent spectrums to show the various behavior patterns of the system. To convert vibrating motion into electrical power, a piezoelectric sensor is connected to the dynamical model, which is just one of the energy harvesting (EH) technologies with extensive applications in the commercial, industrial, aerospace, automotive, and medical industries. Moreover, the time histories of the obtained solutions with and without control are analyzed graphically. Finally, resonance curves are used to discuss stability analysis and steady-state solutions.

**Supplementary Information:**

The online version contains supplementary material available at 10.1038/s41598-024-75342-z.

## Introduction

Vibrations and dynamic instability are common in equipment and structural systems, potentially leading to severe incidents and equipment failures. Consequently, vibration control has emerged as a crucial development in recent decades, particularly for lightweight and low-damped structures, to minimize oscillations and extend the lifespan of these systems by preventing damage or catastrophic events. This topic has garnered significant attention from researchers and scientists due to its impact on equipment, industry, and economic efficiency.

In^1^, time-delayed feedback control for displacement and velocity was developed to enhance the stability of a single DOF giant magnetostrictive actuator system and regulate its nonlinear dynamic characteristics, including primary frequency response, chaotic motion, and limit cycle amplitude. Magnetostrictive materials, a new class of materials, have been widely applied in EH, aviation, electronically controlled fuel injection, and electro-hydraulic servo valves, among other fields^2–7^. In^8^, the authors formulated the nonlinear dynamic differential equation for a cable-stayed beam under time-delayed feedback control. The findings indicated that velocity feedback control was less effective than displacement or acceleration feedback control. In^9^, a torsional vibration model for an electromechanical transmission system was developed, showing that as feedback gain increased, the instability region shrank, leading to a transition from chaotic to periodic motion.

In^10^, the dynamic equation for a transmission system with electromechanical coupling was derived. The study showed that the zero solution remained locally stable with a sufficiently small time delay. However, as the time-delay parameter increased beyond a critical value, the stability of the zero solution changed, leading to bifurcation into periodic solutions. The Van-der-Pol oscillations in feedback state control with time delay, focusing on fundamental resonance, are investigated in^11^. Numerical simulations confirmed that carefully chosen delay feedback values could eliminate modification of motion and reduce the peak amplitude of primary resonance. On the other hand, the worst resonance state in a feedback-controlled, externally stimulated Duffing oscillator is examined in^12^. To derive the AS for the system, as previously mentioned, the MSS is used to assess the presence of time delay in the control loop. Options for feedback outcomes and time delay are explored, including a thorough stability analysis and effective reduction of amplitude peaks. Additionally, the analytical results are compared with numerical results using the RK-4 method. Using a selective negative derivative feedback (NDF) algorithm^13^, investigated a distributed wireless-based control method. In^14^, the performance of NDF and positive position feedback (PPF) for controlling the oscillation of an extensible arm with piezoelectric actuators are evaluated. The study found that NDF controllers outperformed PPF controllers according to the performance measures assessed.

In^15^, the authors address the optimization of a three-element dynamic vibration absorber. This absorber incorporates an inerter—a component designed to counteract vibrations—along with grounded stiffness. The study thoroughly analyzes how various design parameters affect the performance of the vibration absorber. The absorber’s ability to mitigate unwanted vibrations across various operating conditions is improved. The obtained outcomes offer valuable insights into the design and implementation of dynamic vibration absorbers, highlighting the potential for enhanced vibration suppression through carefully tuned parameters. In^16^, the authors explored PPF controllers with robust phase compensators for vibration suppression in wind tunnel support structures, demonstrating enhanced control effectiveness​.

The design process for NDF filtration in combined systems with the H2 or H ∞ approach and maximum dampening is described in^17^. As a band-pass filter, the NDF controller can efficiently eliminate disturbances at lower and higher frequencies. The findings demonstrate that NDF can not only powerfully and effortlessly damp a particular mode, but it can also, without causing any instability, lower some levels of vibrations in the modes that are close to the targeted one. A simple process for designing a NDF controller using the H2 optimization strategy and maximum damping is also provided in^18^. By splitting the action of control away from frequencies naturally connected to the regulated behaviors, NDF, a controller acting as a band-pass filter, lowers spillover impact. Moreover, in^19^, an analytical and experimental evaluation of a distributed NDF controller-based active vibrating reduction technique for bladed constructions with piezoelectric regions was offered. In^20^, the authors presented the actions of a resonant control method on a quarter-vehicle car that is exposed to the principal parametric excitement, known as the NDF controller. As a result, we considered the NDF controller, which is crucial to it.

Additionally, for the first time, the tolerance of a quarter-vehicle transportation system to variations in natural frequency has been examined. The equations governing the controlled system have been mathematically solved using the multiple-scale homotopy technique. Among renewable energy sources, The EH is highlighted as particularly promising for converting ambient waste energy into usable forms^21–23^. In^24^, the authors investigated a three-degree-of-freedom dynamical system, deriving the equations of motion using Lagrangian formulas and analyzing the stability using the MSS. The reliability of the AS results was validated by comparing them with numerical simulations. The study also determined the ME by analyzing resonance conditions and solvability criteria. Bifurcation plots and Lyapunov exponent spectra were used to illustrate various motion forms of the system, with Poincaré maps further detailing the dynamics.

In^25^, the authors developed a dynamical model of the shearer permanent magnetic small drive breaking transmission utilizing the MSS. The multi-excitation force mechanism was examined and resolved. Additionally, the terrible resonance incidences via the cubic velocity feedback (CVF) controller and velocity feedback (VF) controller were discussed. It became clearer how the VFB controller and CVF controller affected each other. It was demonstrated that the control impact is approximately 99.95% attributable to the VF controller result and approximately 96.7% attributable to the CVF controller effect. Because of this, the VF controller performs better for this system when both parametric and external excitation forces are applied. In^26^, a mathematical model of a spinning beam at varied speeds is investigated. The nonlinear equations of the system are subjected to the MSS, which examines the approximate solution of the system’s behavior in the resonance situation. We looked at the system when a proportional-derivative (PD) controller gave displacement and velocity delayed control. In^27^, a study was conducted on a coupled pitch-roll ship model using negative cubic velocity feedback (NCVF) control that responds to parametric excitation. In^28^, the authors compare three controller methods for a cracked beam under harmonic excitation PPF, integral resonant control, and nonlinear integral PPF. The MSS is applied for an approximate solution, and the nonlinear integral PPF controller is the best at decreasing system amplitude. The control of a laminated shell system composed of macro-fiber composite utilizing a PD controller is examined in^29^. The goals are to reduce the vibration caused by the model and improve system stability. Through the capacity to predict future framework response faults, the PD controller reduces peak overshoot, increases stability, and expedites the settlement time. The controller restores the transient responsiveness of the framework by lowering vibration amplitudes. The results show high vibration reduction efficiency when compared to other control algorithms and earlier research. In^30^, the authors examined a dynamic vibration controller that uses Galerkin’s approach and Hamilton’s statue to regulate nonlinear vibrations in a circular truss antenna. The controller decreases oscillations and lessens the motion of the shaft ring structure. Four-dimensional averaged equations were obtained to monitor internal resonances across MSS. The controller’s NS were compared to previous papers, and good agreement was demonstrated with methodical ones.

In recent studies on vibrational dynamics and stability, works^31–35^ have contributed significant insights into various aspects of dynamic systems. The study in^31^ explores the stability and analysis of a 4DOF dynamical system near resonance. This study provides a detailed analysis of how resonance affects system stability and highlights potential approaches to managing these effects. Building on this^32^, examines a 3DOF vibrating system, offering insights into its stability when operating close to resonance conditions. This research further elucidates the complexities of maintaining system stability under various resonant conditions. The vibrational dynamics of systems subjected to external torques and excitation forces are explored in^33^. This work extends the understanding of how external forces impact system behavior, emphasizing the importance of external factors in dynamic analysis. The study in^34^ focuses on a dynamical system equipped with a piezoelectric energy harvester device. The analysis reveals how such devices can influence system stability near resonance, providing valuable information for designing more efficient energy harvesting systems. A comprehensive analysis of a spring pendulum’s forced vibrating planar motion is presented in^35^. However, the complex interactions between the spring pendulum and external forces are investigated, contributing to a deeper understanding of planar dynamic systems.

In this paper, a piezoelectric EH device is connected to a dynamical system to convert vibrational movement into electrical power. The NVF controller minimizes any dangerous vibrations that happen whenever it’s operating in a resonance state. Lagrange’s equations are utilized to derive the principal governing system of motion, while the piezoelectric circuit mechanism is employed to formulate the corresponding equation. The MSS is used to obtain the AS of this system up to the third approximation. Using the RK-4 method, the NS were determined, graphically depicted, and then compared to the AS to assess the accuracy of the perturbation method. After removing the secular terms, the solvability conditions are determined, and all resonance cases are determined. The stability examinations of the ME are solved numerically to show the temporal histories of the modified amplitudes and phases. In the steady-state scenario, these equations are reduced to achieve the frequency response equations, which are solved simultaneously to get the fixed points. Therefore, the related zones of stability and instability are potted through the resonance curves at different values of controller gains. Moreover, the system’s behavior is examined through bifurcation diagrams, Poincaré maps, and Lyapunov exponent spectra. The significance of this study lies in its exploration of the vibrating analysis of a 2DOF dynamical system aimed at generating valuable electrical energy for numerous practical applications through a piezoelectric device.

Therefore, the novelties of this study are highlighted by several innovative aspects in the approach and analysis of a 2DOF dynamical system that incorporates a nonlinear damped harmonic spring pendulum with a piezoelectric device: The introduction of a NVF controller into the system to mitigate undesired vibrations, particularly at resonance, is a key novelty. This approach enhances system efficiency by reducing vibrational impacts, which is crucial for applications where stability and precision are vital. The study uniquely considers the effects of a parametric excitation force along the spring’s elongation direction, coupled with an operating moment at the support point. This combination of forces creates a more complex and realistic model, distinguishing this work from more straightforward dynamical system studies. The use of the MSS to obtain the AS up to the third order is another novel aspect, where this method allows for a more accurate and detailed exploration of the system’s dynamic behavior, particularly in the presence of nonlinearities. The study verifies the accuracy of the AS by comparing them with NS obtained through the RK-4. This dual approach ensures that the analytical methods employed are reliable and robust. The study delves into complex dynamic behaviors using bifurcation diagrams, Poincaré maps, and Lyapunov exponent spectrums. This comprehensive analysis provides new insights into the system’s stability and potential chaotic behavior, which are critical for understanding the full range of system responses. The connection of a piezoelectric sensor to the dynamical model for converting vibrational motion into electrical power is another innovative aspect. This application of energy harvesting technologies is explored within various industries, including commercial, industrial, aerospace, automotive, and medical sectors, demonstrating the research’s practical relevance and potential impact. The graphical analysis comparing the system’s behavior with and without the NVF control provides a visual and quantitative demonstration of the control’s effectiveness, adding a practical dimension to the theoretical outcomes.

## Motivation of this work

The motivation for this study arises from the growing need to optimize the performance of dynamical systems in various industries, particularly in the context of vibration control and energy harvesting. As technological advancements continue to push the boundaries in fields such as aerospace, automotive, and medical industries, the ability to efficiently manage and convert vibrational energy into usable electrical power has become increasingly important. Integrating a piezoelectric device within a 2DOF dynamical system offers a promising avenue for both vibration suppression and energy harvesting.

However, challenges such as unwanted vibrations, especially during resonance, can significantly impair system efficiency. To address this, the study introduces a NVF controller to reduce these detrimental vibrations. By analyzing the system’s behavior under parametric excitation forces and applying advanced analytical methods like the MSS, the research seeks to enhance the understanding of the system’s dynamic responses and stability. The motivation is not only to improve the accuracy and reliability of predictive models through both analytical and NS but also to explore practical applications where energy harvesting technologies can be effectively utilized. The study’s outcomes are expected to contribute to the design of more efficient systems in commercial, industrial, and medical applications, where vibration control and energy harvesting are critical.

## Mathematical analysis of the dynamical model

**Fig. 1:**
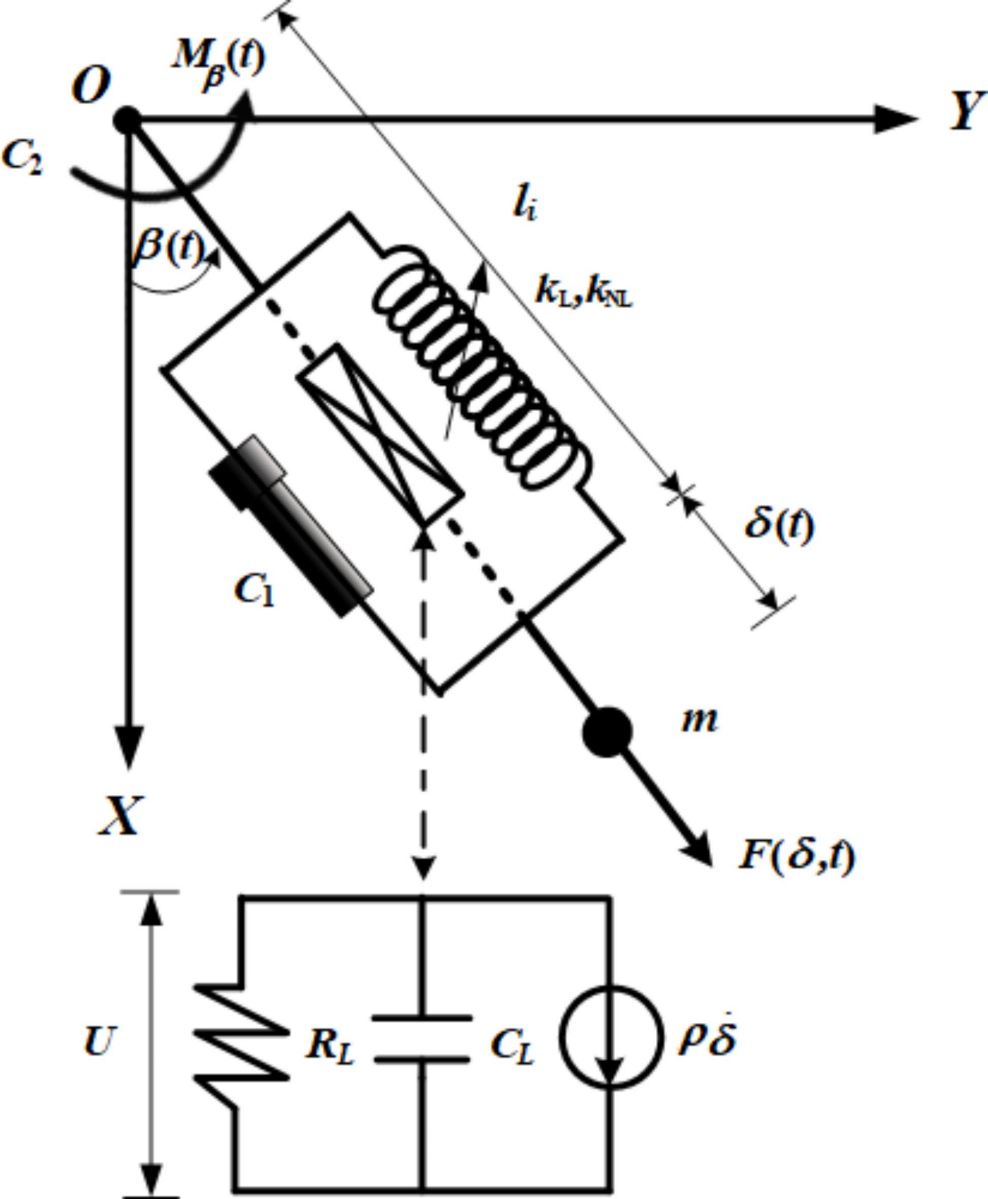
The main system.

**Fig. 2:**
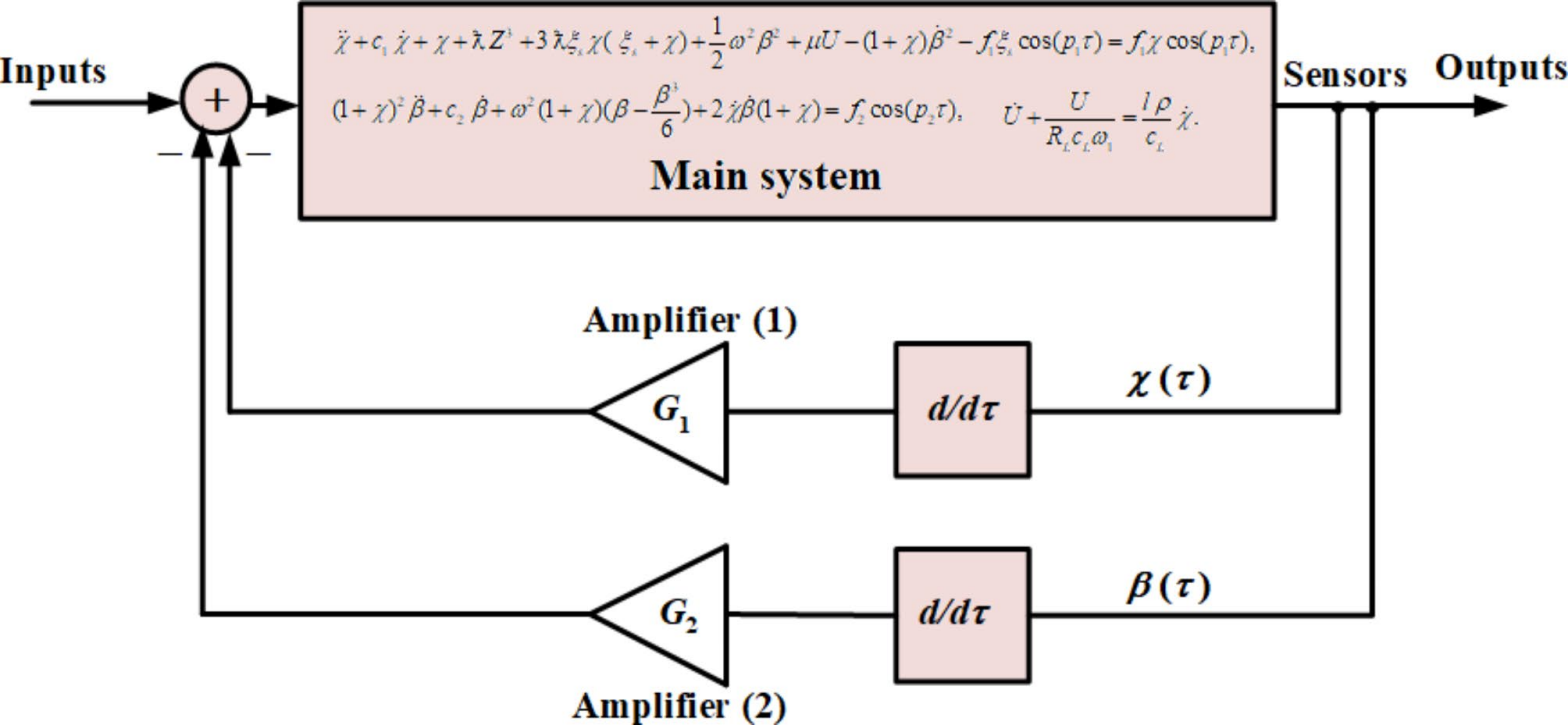
NVF controller of the dynamical model.

This section illustrates the required system under examination, wherein the EOM can be derived in both its primary forms and the related dimensionless forms, in addition to a piezoelectric circuit’s equation. Therefore, let us consider the planar motion of a 2DOF dynamical system consisting of a mass *m* attached to a nonlinear damped harmonic spring pendulum that has a linear and nonlinear stiffness with coefficients ${k_L}$ and ${k_{NL}}$, respectively. As we proceed with describing the relevant system, we consider the parametric external force $F(t,\,\delta )={F^*}\,\delta (t)\cos {\Omega _1}t$ acting on the mass *m* in the spring direction, in addition to the moment ${M_\beta }(t)=M\,\cos {\Omega _2}t\,\,$ operating at *O*. Here $\delta (t)$ signifying the spring elongation, $({F^*},M)$ and $({\Omega _1},\,{\Omega _2})$ are the amplitudes and frequencies of $F(t)$ and ${M_\beta }(t)$, respectively. Let ${C_1}$ and ${C_2}$, respectively, symbolize the damping coefficients of the damping force ${C_1}\dot {\delta }$ and damping moment ${C_2}\dot {\beta }$, *g* is the acceleration due to gravity and ${l_i}$ is the unstretched length of the pendulum. When connecting the piezoelectric device, we need to consider the resistive load of the device’s circuit ${R_L}$. Let ${c_L}$ denotes the capacitance of the piezoelectric and $\rho$ indicates the linear coupled coefficient for the piezoelectric circuit, as demonstrated in Fig. 1. The NVF controller with gains ${G_1}$ and ${G_2}$ was also used, as shown in Fig. 2 to limit the increase in the fluctuation amplitudes during resonance.

The Cartesian coordinates of the mass can be expressed as follows

1 $$X=({l_i}+\delta )\cos \,\beta ,\,\,\,\,\,\,\,\,\,\,Y=({l_i}+\delta )\sin \,\beta .\,$$

The present expressions of the energies of potential and kinetic for the dynamical system are as follows

2 $$\begin{aligned} V & = - mg\,({l_i}+\delta )\cos \,\beta +\frac{1}{2}{k_L}\,{\delta ^2}+\frac{1}{4}{k_{NL}}\,{\delta ^4}, \\ T & =\frac{1}{2}m\{ {[\dot {\delta }\sin \beta +({l_i}+\delta )\dot {\beta }\,\cos \,\beta ]^2}+{[\dot {\delta }\,\cos \beta - ({l_i}+\delta )\dot {\beta }\,\,\sin \,\beta ]^2}\} , \\ \end{aligned}$$

where the differentiation over time *t* is represented by the dot.

The EOM governing the system under investigation are obtained utilizing the subsequent form of Lagrange’s Eq. 

3 $$\frac{d}{{dt}}(\frac{{\partial \Pi }}{{\partial \dot {p}}}) - (\frac{{\partial \Pi }}{{\partial p}})={Q_p};\,\,\,\,\,\,\,\,p=(\delta ,\beta ).$$

Here, $\Pi =T - V$ presents the Lagrangian and ${Q_p}$ indicates the generalized coordinates’ corresponding forces$p=(\delta ,\beta )$, these forces have the forms

4 $$\begin{aligned} {Q_\delta } & =F\,\delta \cos {\Omega _1}t\, - {C_1}\dot {\delta }\, - \rho \,U, \\ {Q_\beta } & =M\,\cos {\Omega _2}t\,\, - {C_2}\dot {\beta }\,, \\ \end{aligned}$$

where *U* is the output voltage of the resistive load ${R_L}$, and $\rho \,$is the coupling coefficient between the harvesting device and the dynamical system.

Furthermore, the piezoelectric circuit’s mechanism equation^35^ can be expressed as follows

5 $${c_L}\,\dot {U}+({U \mathord{\left/ {\vphantom {U {{R_L}}}} \right. \kern-0pt} {{R_L}}}) - \rho \,\dot {\delta }=0.$$

Let’s now explore the next parameters without dimensions

6 $$\begin{aligned} \omega _{1}^{2} & =\frac{{{k_L}\,}}{m},\,\,\,\,\,\omega _{2}^{2}=\frac{{g\,}}{l}\,,\,\,\,\,\,\omega =\frac{{{\omega _2}}}{{{\omega _1}}}\,\,,\,\,\,{p_1}=\frac{{{\Omega _1}}}{{{\omega _1}}},\,\,\,\,{p_2}=\frac{{{\Omega _2}}}{{{\omega _1}}},\,\,\,\,{{^{-}}\!\!\!\!\lambda} =\frac{{{k_{NL}}\,{l^2}}}{{\omega _{1}^{2}m}},\,\,\,\,\mu =\frac{\rho }{{\omega _{1}^{2}m\,\,l}},\,\,\,\, \\ {c_1} & =\frac{{{C_1}}}{{{\omega _1}m}},\,\,\,\,{c_2}=\frac{{{C_2}}}{{{\omega _1}m{l^2}}},\,\,\,\,{f_1}=\frac{F}{{\omega _{1}^{2}ml}},\,\,\,\,\,{f_2}=\frac{M}{{\omega _{1}^{2}m{l^2}}},\,\,\,\,\,\chi =\frac{{\delta - {\delta _s}}}{l},\,\,\,\,\,l={l_i}+{\delta _s},\,\,\,\, \\ {\xi _s} & =\frac{{{\delta _s}}}{l},\,\,{k_L}\,{\delta _s}\,+{k_{NL}}\,\,\delta _{s}^{3}=mg,\,\,\,\,{\xi _s}+\alpha \,\xi _{s}^{3}={\omega ^2},\,\,\,\,\,\tau ={\omega _1}t, \\ \end{aligned}$$

where ${\delta _s}$ is the spring’s static elongation.

When (2), (4), and (6) are substituted into (3), the governing EOM in dimensionless forms below with control is obtained.

7 $$\begin{aligned} & \ddot {\chi }+{c_1}\,\dot {\chi }+{G_1}\,\dot {\chi }+\chi +{{^{-}}\!\!\!\!\lambda} \,{\chi ^3}+3\,{{^{-}}\!\!\!\!\lambda} {\xi _s}\chi (\,\,{\xi _s}+\chi )+\frac{1}{2}{\omega ^2}{\beta ^2}+\mu U - (1+\chi ){{\dot {\beta }}^2} \\ & \quad - {f_1}{\xi _s}\cos ({p_1}\tau )={f_1}\chi \cos ({p_1}\tau ), \\ \end{aligned}$$

8 $${(1+\chi )^2}\ddot {\beta }+{c_2}\,\dot {\beta }+{G_2}\,\dot {\beta }+{\omega ^2}(1+\chi )(\beta - \frac{{{\beta ^3}}}{6})+2\dot {\chi }\dot {\beta }(1+\chi )={f_2}\cos ({p_2}\tau ).$$

Using the previously mentioned procedure, we can determine the dimensionless equation of the piezoelectric circuit by substituting (6) into (5), as illustrated below

9 $$\dot {U}+\frac{U}{{{R_L}{c_L}{\omega _1}}}=\frac{{l\,\rho }}{{{c_L}}}\dot {\chi }.$$

It must be noted that for vibrating dynamical systems, setting parameters in dimensionless forms is particularly important for the following reasons:

These forms often simplify the DEs governing the vibrating system, making AS and numerical ones more tractable. In such systems, dimensionless parameters can help identify key resonance conditions where the system’s response is maximized. This is crucial for designing systems to avoid or exploit resonance. Furthermore, dimensionless parameters enable the scaling of results. This means outcomes from a model system can be applied to real-world systems of different sizes or materials, making the research broadly applicable. These parameters allow for easier comparison between different vibrating systems or configurations, helping to identify optimal designs or configurations for specific applications.

## The proposed methodology

The AS of the previously given system of Eqs. (7)-(9) is obtained in the present part using the MSS. As a result, we concentrate our research on this system’s dynamic behavior in a small area enclosed by the static equilibrium point^36,37^. Then, its vibrational amplitudes can be expressed as follows

10 $$\chi (\tau )=\varepsilon \,\tilde {\gamma }(\tau ;\varepsilon ),\,\,\,\,\beta (\tau )=\varepsilon \,\tilde {\psi }(\tau ;\varepsilon ),\,\,\,\,\,U(\tau )=\varepsilon \,\tilde {\upsilon }(\tau ;\varepsilon ),$$

where $0<\varepsilon <<1$ is a small parameter. The smallness of the variables and parameters was taken into account to correspond with

11 $$\begin{aligned} \mu & ={\varepsilon ^2}\,\tilde {\mu },\,\,\,\,\,{c_1}=\,{\varepsilon ^2}\,{{\tilde {c}}_1},\,\,\,\,\,{c_2}=\,{\varepsilon ^2}\,{{\tilde {c}}_2},\,\,\,\,\,{G_1}=\,{\varepsilon ^2}\,{{\tilde {G}}_1},\,\,\,\,\,{G_2}=\,{\varepsilon ^2}\,{{\tilde {G}}_2},\,\,\,\,\,\,{{^{-}}\!\!\!\!\lambda} ={\varepsilon ^2}\,\tilde {{^{-}}\!\!\!\!\lambda},\, \\ \rho & ={\varepsilon ^2}\,\tilde {\rho },\,\,\,\,\,{\xi _s}=\varepsilon {{\tilde {\xi }}_s},\,\,\,\,\,{R_L}={{{{\tilde {R}}_L}} \mathord{\left/ {\vphantom {{{{\tilde {R}}_L}} {{\varepsilon ^2}}}} \right. \kern-0pt} {{\varepsilon ^2}}},\,\,\,\,\,{c_L}\,={\varepsilon ^2}{{\tilde {c}}_L},\,\,\,\,\,{f_1}=\,{\varepsilon ^2}\,{{\tilde {f}}_1}\,,\,\,\,\,\,{f_2}=\,{\varepsilon ^3}\,{{\tilde {f}}_2}.\, \\ \end{aligned}$$

In alignment with the MSS approach, the solutions $\tilde {\gamma },\,\,\tilde {\psi },$ and $\tilde {\upsilon }$ can be stated as illustrated below^38,39^

12 $$\begin{aligned} \tilde {\gamma } & =\sum\nolimits_{{k=1}}^{3} {{\varepsilon ^k}{\gamma _k}({\tau _0},\,{\tau _1},\,{\tau _2})} +O({\varepsilon ^4}), \\ \tilde {\psi } & =\sum\nolimits_{{k=1}}^{3} {{\varepsilon ^k}{\psi _k}({\tau _0},\,{\tau _1},\,{\tau _2})} +O({\varepsilon ^4}), \\ \tilde {\upsilon } & =\sum\nolimits_{{k=1}}^{3} {{\varepsilon ^k}{\upsilon _k}({\tau _0},\,{\tau _1},\,{\tau _2})} +O({\varepsilon ^4}). \\ \end{aligned}$$

Here, ${\tau _n}={\varepsilon ^n}\tau \,\,(n=0,\,1\,,\,2)$ reflect specific time scales, ${\tau _1}$ represents the fast scale, while ${\tau _2}$ and ${\tau _3}$ denote the slower ones. It is now necessary to alter derivatives in relation to $\tau$ into these scales. Thus, the following differential operators can be used to accomplish this objective

13 $$\begin{aligned} \frac{d}{{d\tau }} & =\frac{\partial }{{\partial {\tau _0}}}+\varepsilon \frac{\partial }{{\partial {\tau _1}}}+{\varepsilon ^2}\frac{\partial }{{\partial {\tau _2}}}, \\ \frac{{{d^2}}}{{d{\tau ^2}}} & =\frac{{{\partial ^2}}}{{\partial \tau _{0}^{2}}}+2\varepsilon \frac{{{\partial ^2}}}{{\partial {\tau _0}\partial {\tau _1}}}+{\varepsilon ^2}(\frac{{{\partial ^2}}}{{\partial \tau _{1}^{2}}}+2\frac{{{\partial ^2}}}{{\partial {\tau _0}\partial {\tau _2}}})+O({\varepsilon ^3}). \\ \end{aligned}$$

These operators make it evident that terms $O({\varepsilon ^3})$ and higher are not considered due to their small quantity. The following sets of partial DEs are produced when (10)-(13) are substituted into Eqs. (7)-(9). These partial DEs are connected to the different powers of $\varepsilon$

(i) Order of $$\varepsilon$$

14 $$\frac{{{\partial ^2}{\gamma _1}}}{{\partial \tau _{0}^{2}}}+{\gamma _1}=0,$$

15 $$\frac{{{\partial ^2}{\psi _1}}}{{\partial \tau _{0}^{2}}}+{\omega ^2}{\psi _1}=0,\,$$

16 $$\frac{{\partial {\upsilon _1}}}{{\partial {\tau _0}}}+\frac{{{\upsilon _1}}}{{{{\tilde {c}}_L}{{\tilde {R}}_L}{\omega _1}}}=\frac{{l\,\tilde {\rho }}}{{{{\tilde {c}}_L}}}\frac{{\partial {\gamma _1}}}{{\partial {\tau _0}}}.$$

(ii) Order of ${\varepsilon ^2}$

17 $$\frac{{{\partial ^2}{\gamma _2}}}{{\partial \tau _{0}^{2}}}+{\gamma _2}= - \frac{1}{2}{\omega ^2}\psi _{1}^{2}+{(\frac{{\partial {\psi _1}}}{{\partial {\tau _0}}})^2} - 2\frac{{{\partial ^2}{\gamma _1}}}{{\partial {\tau _0}\partial {\tau _1}}},$$

18 $$\frac{{{\partial ^2}{\psi _2}}}{{\partial \tau _{0}^{2}}}+{\omega ^2}{\psi _2}= - 2(\frac{{{\partial ^2}{\psi _1}}}{{\partial {\tau _0}{\tau _1}}}\,+\frac{{\partial {\gamma _1}}}{{\partial {\tau _0}}}\frac{{\partial {\psi _1}}}{{\partial {\tau _0}}}) - {\gamma _1}({\omega ^2}{\psi _1}+2\frac{{{\partial ^2}{\psi _1}}}{{\partial \tau _{0}^{2}}}),$$

19 $$\frac{{\partial {\upsilon _2}}}{{\partial {\tau _0}}}+\frac{{{\upsilon _2}}}{{{{\tilde {c}}_L}{{\tilde {R}}_L}{\omega _1}}}=\frac{{l\,\tilde {\rho }}}{{{{\tilde {c}}_L}}}(\frac{{\partial {\gamma _1}}}{{\partial {\tau _1}}}+\frac{{\partial {\gamma _2}}}{{\partial {\tau _0}}}) - \frac{{\partial {\upsilon _1}}}{{\partial {\tau _1}}}.$$

(iii) Order of ${\varepsilon ^3}$

20 $$\begin{aligned} \frac{{{\partial ^2}{\gamma _3}}}{{\partial \tau _{0}^{2}}}+{\gamma _3} & =\frac{1}{2}{{\tilde {f}}_1}{{\tilde {\xi }}_s}{e^{i\,{p_1}\,{\tau _0}}}+\frac{1}{2}{{\tilde {f}}_1}{\gamma _1}{e^{i\,{p_1}\,{\tau _0}}} - {\omega ^2}{\psi _1}{\psi _2} - {{\tilde {c}}_1}\frac{{\partial {\gamma _1}}}{{\partial {\tau _0}}} - {{\tilde {G}}_1}\frac{{\partial {\gamma _1}}}{{\partial {\tau _0}}} - \frac{{{\partial ^2}{\gamma _1}}}{{\partial \tau _{1}^{2}}} \\ & \quad +2(\frac{{\partial {\psi _1}}}{{\partial {\tau _0}}}\frac{{\partial {\psi _1}}}{{\partial {\tau _1}}}+\frac{{\partial {\psi _1}}}{{\partial {\tau _0}}}\frac{{\partial {\psi _2}}}{{\partial {\tau _0}}}) - 2(\frac{{{\partial ^2}{\gamma _1}}}{{\partial {\tau _0}\partial {\tau _2}}}+\frac{{{\partial ^2}{\gamma _2}}}{{\partial {\tau _0}\partial {\tau _1}}})+\,{\gamma _1}{(\frac{{\partial {\psi _1}}}{{\partial {\tau _0}}})^2} - \tilde {\mu }\,{\upsilon _1}, \\ \end{aligned}$$

21 $$\begin{aligned} \frac{{{\partial ^2}{\psi _3}}}{{\partial \tau _{0}^{2}}}+{\omega ^2}{\psi _3} & =\frac{1}{2}{{\tilde {f}}_2}{e^{i\,{p_2}\,{\tau _0}}} - {{\tilde {c}}_2}\frac{{\partial {\psi _1}}}{{\partial {\tau _0}}} - {{\tilde {G}}_2}\frac{{\partial {\psi _1}}}{{\partial {\tau _0}}} - 2(\frac{{{\partial ^2}{\psi _1}}}{{\partial {\tau _0}\partial {\tau _2}}}+\frac{{{\partial ^2}{\psi _2}}}{{\partial {\tau _0}\partial {\tau _1}}}) - \frac{{{\partial ^2}{\psi _1}}}{{\partial \tau _{1}^{2}}} \\ & \quad - {\omega ^2}({\psi _2}{\gamma _1}+{\psi _1}{\gamma _2})\,+\frac{1}{6}{\omega ^2}\psi _{1}^{3} - 2{\gamma _1}(\frac{{\partial {\gamma _1}}}{{\partial {\tau _0}}}\frac{{\partial {\psi _1}}}{{\partial {\tau _0}}}+2\frac{{{\partial ^2}{\psi _1}}}{{\partial {\tau _0}\partial {\tau _1}}}+\frac{{{\partial ^2}{\psi _2}}}{{\partial \tau _{0}^{2}}}) \\ & \quad - 2\frac{{\partial {\gamma _1}}}{{\partial {\tau _0}}}(\frac{{\partial {\psi _1}}}{{\partial {\tau _1}}}+\frac{{\partial {\psi _2}}}{{\partial {\tau _0}}}) - 2\frac{{\partial {\psi _1}}}{{\partial {\tau _0}}}(\frac{{\partial {\gamma _1}}}{{\partial {\tau _1}}}+\frac{{\partial {\gamma _2}}}{{\partial {\tau _0}}}) - \frac{{{\partial ^2}{\psi _1}}}{{\partial \tau _{0}^{2}}}(\gamma _{1}^{2}+2{\gamma _2}), \\ \end{aligned}$$

22 $$\frac{{\partial {\upsilon _3}}}{{\partial {\tau _0}}}+\frac{{{\upsilon _3}}}{{{{\tilde {c}}_{_{L}}}{{\tilde {R}}_L}{\omega _1}}}=\frac{{l\,\tilde {\rho }}}{{{{\tilde {c}}_L}}}(\frac{{\partial {\gamma _3}}}{{\partial {\tau _0}}}+\frac{{\partial {\gamma _2}}}{{\partial {\tau _1}}}+\frac{{\partial {\gamma _1}}}{{\partial {\tau _2}}}) - \frac{{\partial {\upsilon _2}}}{{\partial {\tau _1}}} - \frac{{\partial {\upsilon _1}}}{{\partial {\tau _2}}}.$$

It is possible to solve the preceding Eqs. (14)-(22) in a sequential fashion, which highlights the significance of the solutions offered by the first set of Eqs. (14)-(16). Hence, the general solutions to equations (13) to (15) are as follows

23 $${\gamma _1}\,={E_1}\,{e^{i{\tau _0}}}+{\bar {E}_1}\,{e^{ - i{\tau _0}}},$$

24 $${\psi _1}={E_2}\,{e^{i\omega {\tau _0}}}+{\bar {E}_2}{e^{ - i\omega {\tau _0}}},$$

25 $${v_1}={\tilde {R}_L}\tilde {\rho }{\omega _1}l\{ \frac{{{E_1}{e^{i{\tau _0}}}}}{{{{\tilde {R}}_L}{{\tilde {c}}_L}{\omega _1} - i}}+\frac{{{{\bar {E}}_1}{e^{ - i{\tau _0}}}}}{{{{\tilde {R}}_L}{{\tilde {c}}_L}{\omega _1}+i}}\} .$$

Here, ${E_a}\,\,(a=1,2)$ depict obscure complicated procedures in operation at slow time scales ${\tau _a}$, while ${\bar {E}_a}$ show their complex conjugate. The higher-order partial DEs (17)-(19) can be solved by substituting the prior solutions (23)-(25) to get the secular terms. To eliminate these terms, the following conditions can be used

26 $$\frac{{\partial {E_1}}}{{\partial {\tau _1}}}=0,\,\,\,\,\,\frac{{\partial {E_2}}}{{\partial {\tau _1}}}=0.\,$$

Thus, the following solutions are the second-order approximation

27 $${\gamma _2}=\,{\omega ^2}{E_2}{\bar {E}_2}+\frac{{3{\omega ^2}E_{2}^{2}\,}}{{(8{\omega ^2} - 2)}}{e^{2i\omega {\tau _0}}}+CC,\,$$

28 $${\psi _2}= - \frac{{\omega (\omega +2){E_1}\,{E_2}\,}}{{(2\,\omega +1)}}{e^{i(\omega +1){\tau _0}}}+\frac{{\omega (\omega - 2){{\bar {E}}_1}\,{E_2}}}{{(2\,\omega - 1)}}\,{e^{i(\omega - 1){\tau _0}}}+CC,$$

29 $${\upsilon _2}\,=\frac{{3{\omega ^3}\tilde {\rho }l\,{{\tilde {R}}_L}\,{\omega _1}\,E_{2}^{2}}}{{(4\,{\omega ^2} - 1)(2{{\tilde {c}}_L}{{\tilde {R}}_L}\omega {\omega _1} - i)}}{e^{2i\omega {\tau _0}}}+CC\,,$$

where $CC$ shows the previous expressions’ conjugations. In keeping with the case above, the cancellation of the secular terms demanded that ${E_a}$ depended on ${\tau _2}$. Consequently, the following conditions lead to eliminating these terms

30 $$- 2i\frac{{\partial {E_1}}}{{\partial {\tau _2}}} - i(\,{\tilde {c}_1}+{\tilde {G}_1}){E_1} - {H_1}{E_1}+{H_2}{E_1}\,{E_2}\,{\bar {E}_2}=0,$$

31 $$- 2i\omega \frac{{\partial {E_2}}}{{\partial {\tau _2}}} - i\omega \,({\tilde {c}_2}+{\tilde {G}_2}){E_2}+{H_2}{E_1}{E_2}{\bar {E}_1}+{H_3}E_{2}^{2}{\bar {E}_2}=0.$$

In the final stages, we get the third-order solutions, as given below

32 $$\begin{aligned} {\gamma _3} & =\frac{{{{\tilde {f}}_1}{{\tilde {\xi }}_s}{e^{i\,{p_1}{\tau _0}}}}}{{2(1 - p_{1}^{2})}}\,+\frac{{{{\tilde {f}}_1}{E_1}{e^{i\,({p_1}+1){\tau _0}}}}}{{2[1 - {{({p_1}+1)}^2}]}}\,+\frac{{{{\tilde {f}}_1}{{\bar {E}}_1}{e^{i\,({p_1} - 1){\tau _0}}}}}{{2[1 - {{({p_1} - 1)}^2}]}}\,+\frac{{{H_4}{E_1}E_{2}^{2}{e^{i{\text{(}}2\omega +1{\text{)}}{\tau _0}}}}}{{{\text{[}}1 - {{{\text{(}}2\omega +1{\text{)}}}^2}{\text{]}}}}\, \\ & \quad +\frac{{{H_5}{{\bar {E}}_1}E_{2}^{2}{e^{i{\text{(}}2\omega - 1{\text{)}}{\tau _0}}}}}{{{\text{[}}1 - {{{\text{(}}2\omega - 1{\text{)}}}^2}{\text{]}}}}+CC, \\ \end{aligned}$$

33 $${\psi _3}=\frac{{{{\tilde {f}}_2}{e^{i\,{p_2}{\tau _0}}}}}{{2({\omega ^2} - p_{2}^{2})}} - \frac{{\,{H_6}\,E_{1}^{2}{E_2}{e^{i(\omega +2){\tau _0}}}}}{{4(\omega +1)}}+\frac{{\,\,{H_7}\bar {E}_{1}^{2}{E_2}{e^{i(\omega - 2){\tau _0}}}}}{{4(\omega - 1)}} - \frac{{\,\,{H_8}E_{2}^{3}{e^{3\,i\,\omega {\tau _0}}}}}{{8{\omega ^2}}}+CC,$$

34 $$\begin{aligned} {\upsilon _3} & =\frac{{i\,{{\tilde {\xi }}_s}{\omega _1}\,{{\tilde {R}}_L}\,l\,\tilde {\rho }\,{p_1}{{\tilde {f}}_1}{e^{i\,{p_1}{\tau _0}}}}}{{2(1+i\,{\omega _1}{p_1}{{\tilde {c}}_L}\,{{\tilde {R}}_L})(1 - p_{1}^{2})}} - \frac{{{E_1}{\omega _1}\,{{\tilde {R}}_L}\,l\,\tilde {\rho }({p_1}+1){{\tilde {f}}_1}{e^{i\,({p_1}+1){\tau _0}}}}}{{2{p_1}({p_1}+2)[ - i\,+{\omega _1}({p_1}+1){{\tilde {c}}_L}\,{{\tilde {R}}_L}]}} \\ & \quad - \frac{{{{\bar {E}}_1}{\omega _1}\,{{\tilde {R}}_L}\,l\,\tilde {\rho }({p_1} - 1){{\tilde {f}}_1}{e^{i\,({p_1} - 1){\tau _0}}}}}{{2{p_1}({p_1} - 2)[ - i\,\,+{\omega _1}({p_1} - 1){{\tilde {c}}_L}\,{{\tilde {R}}_L}]}} - \frac{{l\,\tilde {\rho }{E_1}E_{2}^{2}{H_4}{\omega _1}\,{{\tilde {R}}_L}\,(2\omega +1){{\tilde {f}}_1}{e^{i\,(2\omega +1){\tau _0}}}}}{{4\omega (\omega +1)[ - i\,\,+{\omega _1}(2\omega +1){{\tilde {c}}_L}\,{{\tilde {R}}_L}]}}\, \\ & \quad - \frac{{l\,\tilde {\rho }{{\bar {E}}_1}E_{2}^{2}{H_5}{\omega _1}\,{{\tilde {R}}_L}\,(2\omega - 1){{\tilde {f}}_1}{e^{i\,(2\omega - 1){\tau _0}}}}}{{4\omega (\omega - 1)[ - i\,\,+{\omega _1}(2\omega - 1){{\tilde {c}}_L}\,{{\tilde {R}}_L}]}}+CC. \\ \end{aligned}$$

Here, ${H_j}\,(j=1,2,3,\ldots, 8)$ are mentioned in Appendix (I).

## Classification of resonance and modulation equations

The objectives of this part are to classify the resonance cases, deal with one such case and get the ME. Such conditions may manifest if any of the denominators in the last two solutions approaches vanish^40,41^. As a result, the subsequent opportunities develop.

- Sub-harmonic resonance case is obtained at ${p_1}=2,$
- Primary external resonance case is obtained at ${p_1}=0,\,\,{p_1}=1,\,\,{p_2}=\omega ,$
- Internal resonance case will be satisfied at $$\omega = \pm 0.5,$$$$\omega = \pm 1,$$$$\omega =0.$$

If any of the previously mentioned criteria for resonance are fulfilled, the system under examination will exhibit highly complex behavior. It’s crucial to remember that the solutions generated are still suitable even while the oscillations are far from resonance. Now, let’s take a closer look at the problem and examine the worst-case resonance, which is a combination of the subharmonic and primary external resonance that occurred simultaneously, i.e., ${p_1} \approx 2,\,\,{p_2} \approx \omega$. Then, one can consider the detuning parameters ${\sigma _a}\,\,(a=1,\,2)$ that contribute to achieving this objective, which indicates the closeness of ${p_1}$ and ${p_2}$ to 2 and $\omega$, respectively. Therefore, one writes:

35 $${p_1}=2+{\sigma _1},\,\,\,\,{p_2}=\omega +{\sigma _2},$$

where ${\sigma _a}={\varepsilon ^2}{\tilde {\sigma }_a}\,\,(a=1,2)$. Another way to think of detuning parameters is as a distance from the strict resonance and vibrations^42^. Removing secular terms allows one to get the conditions of solvability. Consequently, the following equations calculate the probability that the conditions are satisfied

36 $$\begin{aligned} & \frac{1}{2}{{\tilde {f}}_1}{{\bar {E}}_1}\,{e^{i{\tau _1}{{\tilde {\sigma }}_1}}} - 2i\frac{{\partial {E_1}}}{{\partial {\tau _2}}} - i(\,{{\tilde {c}}_1}+{{\tilde {G}}_1}){E_1} - {H_1}{E_1}+{H_2}{E_1}\,{E_2}\,{{\bar {E}}_2}=0, \\ & \frac{1}{2}{{\tilde {f}}_2}\,{e^{i{\tau _1}{{\tilde {\sigma }}_2}}} - 2i\omega \frac{{\partial {E_2}}}{{\partial {\tau _2}}} - i\omega \,({{\tilde {c}}_2}+{{\tilde {G}}_2}){E_2}+{H_2}{E_1}{E_2}{{\bar {E}}_1}+{H_3}E_{2}^{2}{{\bar {E}}_2}=0. \\ \end{aligned}$$

The solvability conditions (26) and (36) that have been defined contain four nonlinear partial DEs with respect to ${E_a}$ that depend on ${\tau _2}$. Consequently, we are capable of these functions’ polar form as:

37 $${E_a}({\tau _2})=\frac{{{{\tilde {q}}_a}({\tau _2})}}{2}{e^{i{\Psi _a}({\tau _2})}};\,\,\,\,\,{q_a}=\varepsilon {\tilde {q}_a}\,\,\,\,(a=1,2).$$

Since the functions ${E_a}$ don’t depend on the scales ${\tau _0}$ and ${\tau _1}$, the first-order derivative operator can be made simpler in the way as follows

38 $$\frac{{\partial {E_a}}}{{\partial {\tau _0}}}=\frac{{\partial {E_a}}}{{\partial {\tau _1}}}=0,\,\,\,\,\,\,\,\,\,\,\frac{{\partial {E_a}}}{{\partial \tau }}={\varepsilon ^2}\frac{{\partial {E_a}}}{{\partial {\tau _2}}}.$$

With regard to (38), the solvability requirements of PDE (36) can be transformed into ordinary DEs with the use of subsequent modified phases.

39 $${\eta _1}({\tau _1},{\tau _2})={\tau _1}\,{\tilde {\sigma }_1} - 2{\Psi _1}({\tau _2}),\,\,\,\,\,\,\,{\eta _2}({\tau _1},{\tau _2})={\tau _1}\,{\tilde {\sigma }_2} - {\Psi _2}({\tau _2}).$$

Based on the above, the system’s below can be obtained by substituting (37)-(39) into (36) and splitting the real and imaginary components

40 $$\begin{aligned} {q_1}\frac{{d{\eta _1}}}{{d\tau }} & ={q_1}({\sigma _1} - {H_9})+\frac{{{H_2}}}{4}{q_1}q_{2}^{2}+\frac{1}{2}{f_1}{q_1}\cos {\eta _1}, \\ \frac{{d{q_1}}}{{d\tau }} & = - \frac{1}{2}{q_1}({c_1}\,+{G_1}+{H_{10}})+\frac{1}{4}{f_1}{q_1}\sin {\eta _1}, \\ {q_2}\frac{{d{\eta _2}}}{{d\tau }} & ={q_2}{\sigma _2}+\frac{1}{{8\omega }}{q_2}({H_3}q_{2}^{2}+{H_2}q_{1}^{2})+\frac{1}{{2\omega }}{f_2}\cos {\eta _2}, \\ \frac{{d{q_2}}}{{d\tau }} & = - \frac{1}{2}{q_2}({c_2}+{G_2})+\frac{1}{{2\omega }}{f_2}\sin {\eta _2}. \\ \end{aligned}$$

Here, ${H_9}$ and ${H_{10}}$ are expressed in Appendix (I). Regarding the above modulation’s system (40), one can solve it numerically using the RK-4 method to display the time-dependent behavior of the functions $\delta ,\,\beta$ and *U*, and their related phase planes $\delta \dot {\delta }$ and $\beta \dot {\beta }$ at resonance without and with control. As a result, the below data and initial conditions are considered

$$\begin{aligned} \omega & =0.22,\,\,\,\,\,\mu =0.0001,\,\,\,\,\,{\zeta _s}=0.05,\,\,\,\,\,{f_1}=0.02,\,\,\,\,\,{f_2}=0.01,\,\,\,\,\,\,{c_1}=0.000003,\,\,\,\,\, \\ {c_2} & =0.000004,\,\,\,\,\,{p_1}=2,\,\,\,\,\,{p_2}=\omega ,\,\,\,\,\,{q_1}(0)=0.01,\,\,\,\,\,{q_2}(0)=0.02\,,\,\,\,\,\,{\eta _1}(0)={\eta _2}(0)=0. \\ \end{aligned}$$

**Fig. 3:**
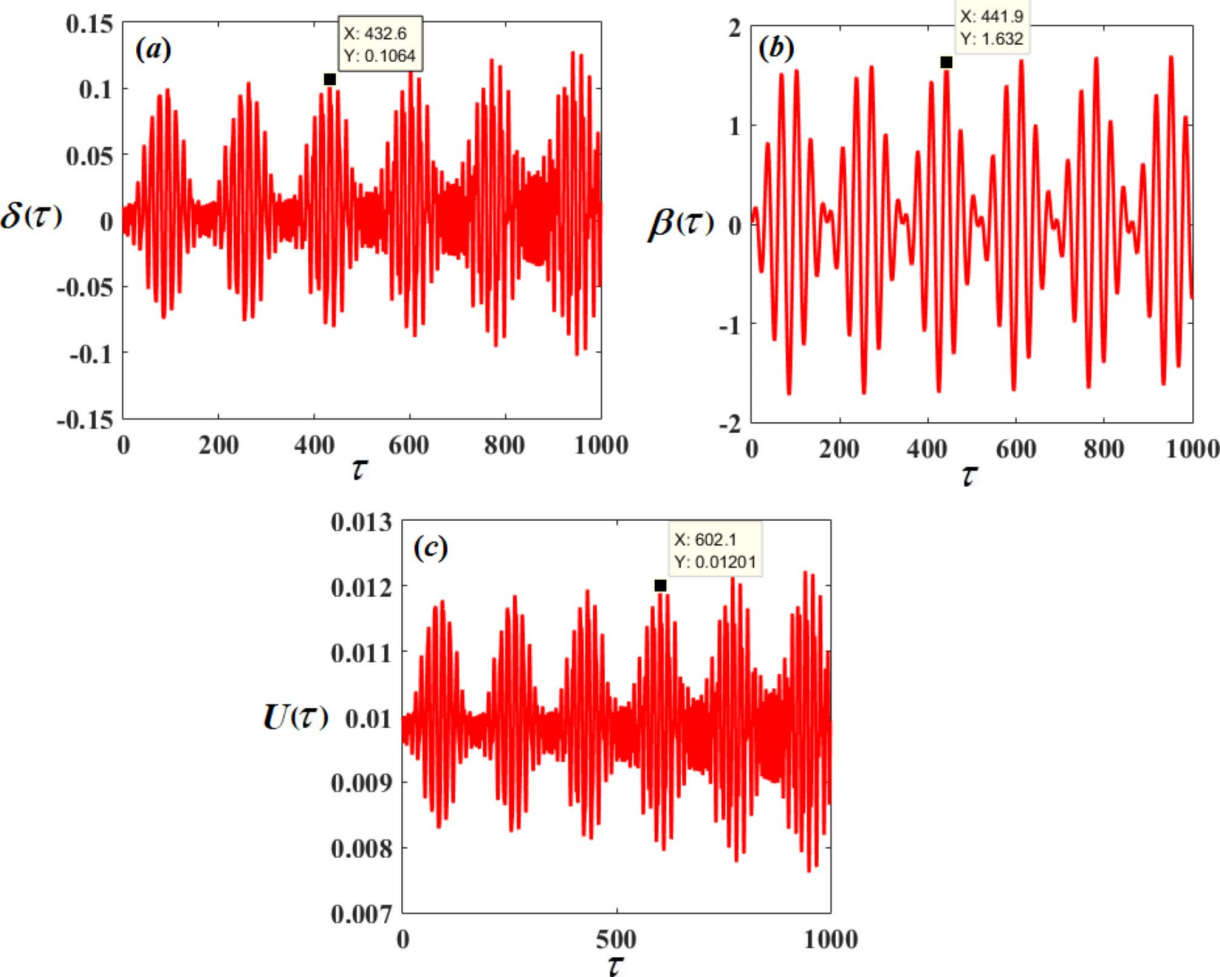
The time response solutions $\delta (\tau ),\,\beta (\tau )$and $U(\tau )$ with resonance cases ${p_1} \approx 2,\,\,{p_2} \approx \omega$without control (${G_1}=0,\,{G_2}=0$).

**Fig. 4:**
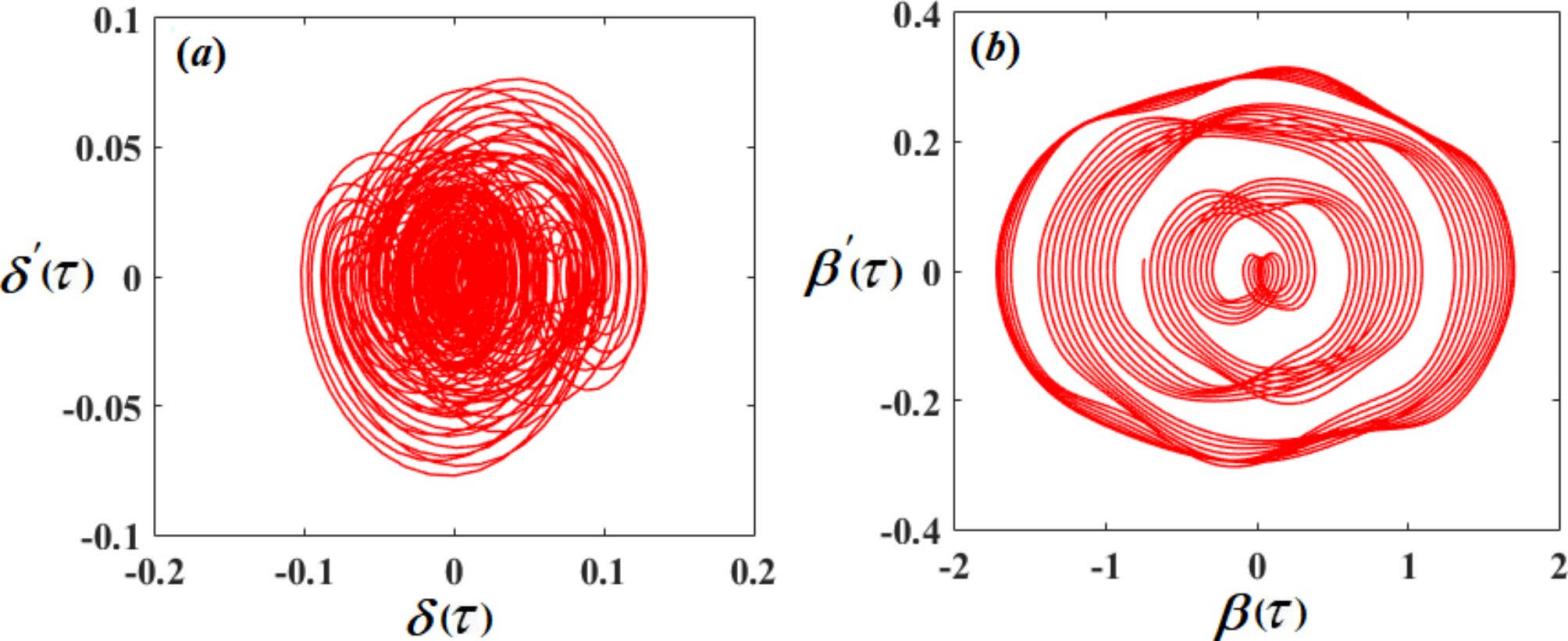
The phase portraits $\delta \,\delta ^{\prime}$ and $\beta \beta ^{\prime}$ with resonance cases ${p_1} \approx 2,\,\,{p_2} \approx \omega$ without control (${G_1}=0,\,{G_2}=0$).

**Fig. 5:**
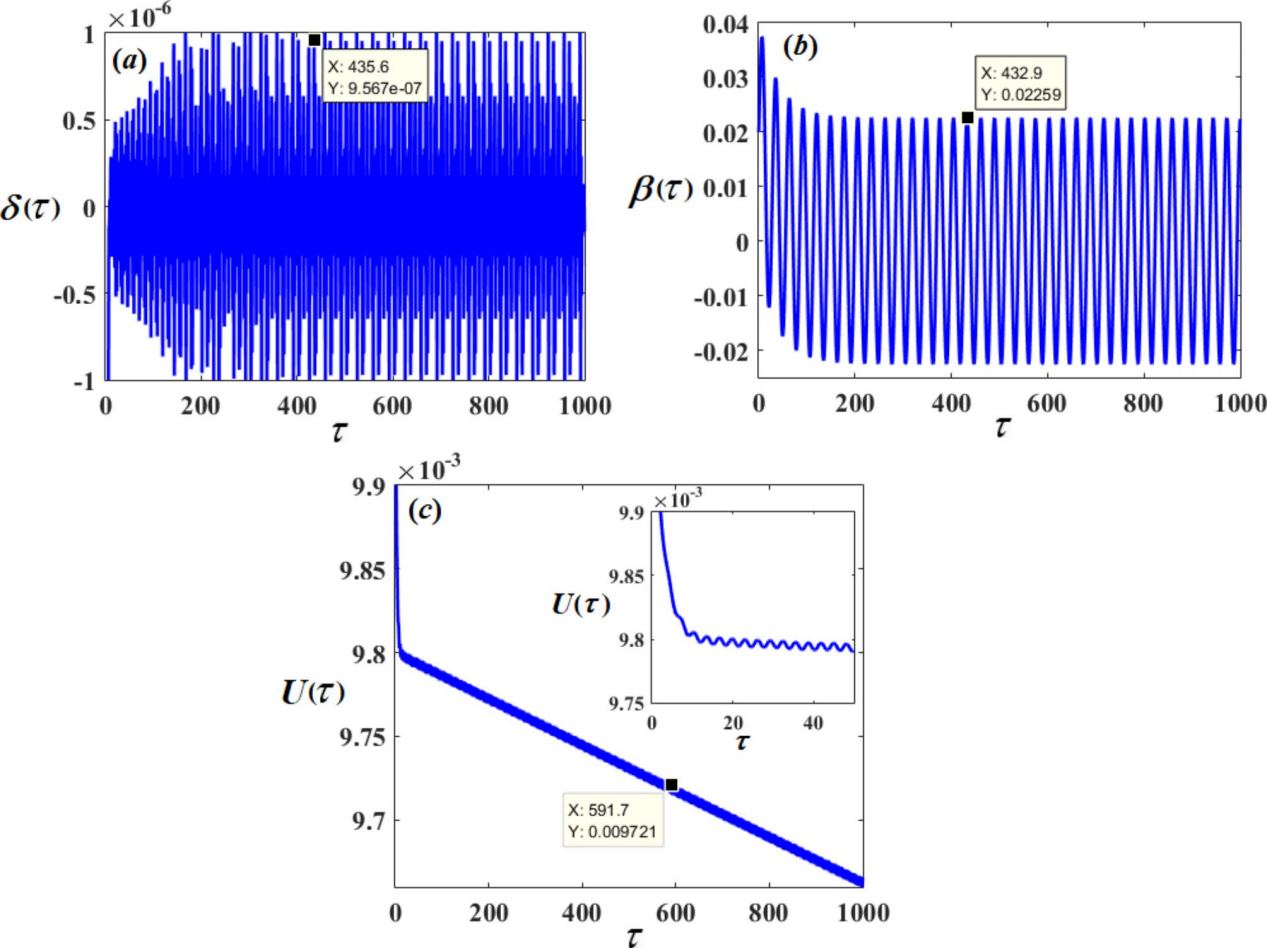
The time response solutions $\delta (\tau ),\,\beta (\tau )$and $U(\tau )$ with resonance cases ${p_1} \approx 2,\,\,{p_2} \approx \omega$with control (${G_1}=3,\,{G_2}=2$).

**Fig. 6:**
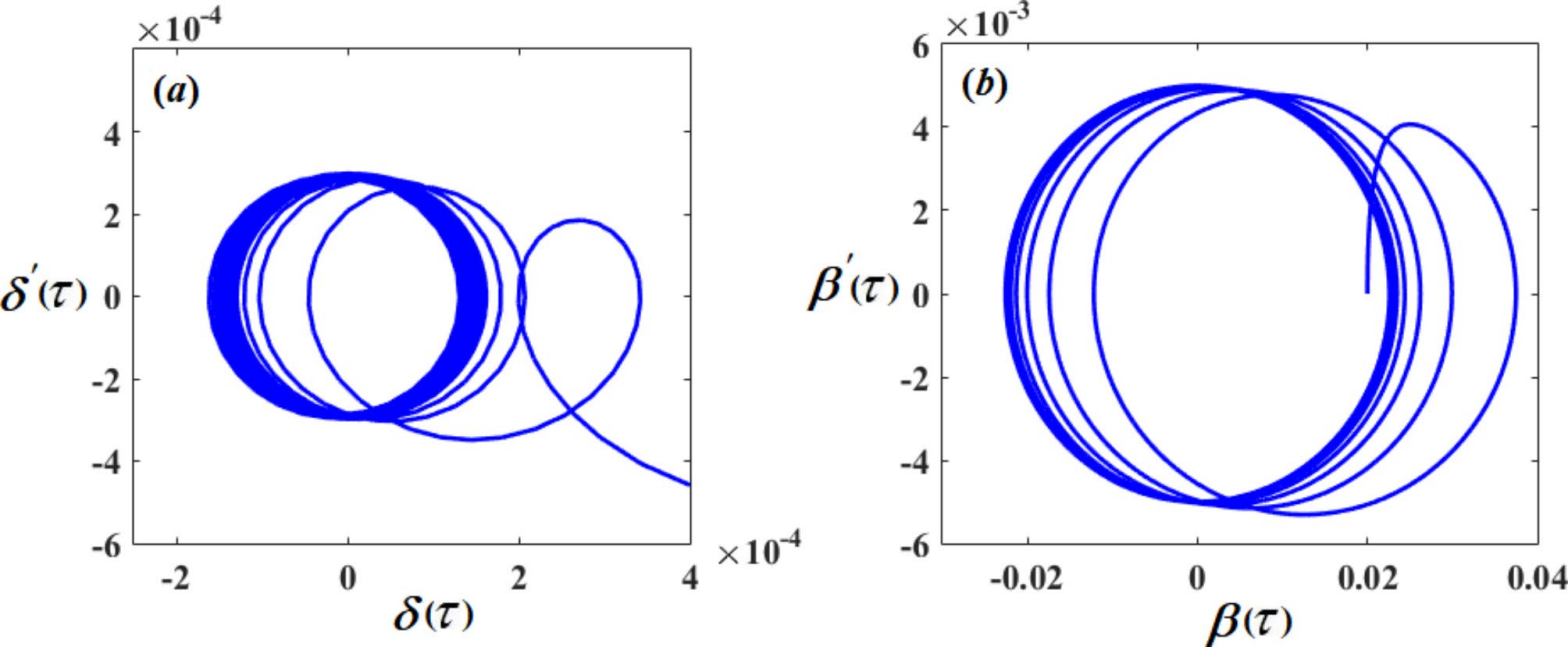
The phase portraits $\delta \,\delta ^{\prime}$ and $\beta \beta ^{\prime}$ with resonance cases ${p_1} \approx 2,\,\,{p_2} \approx \omega$ with control $({G_1}=3,\,{G_2}=2).$

**Fig. 7:**
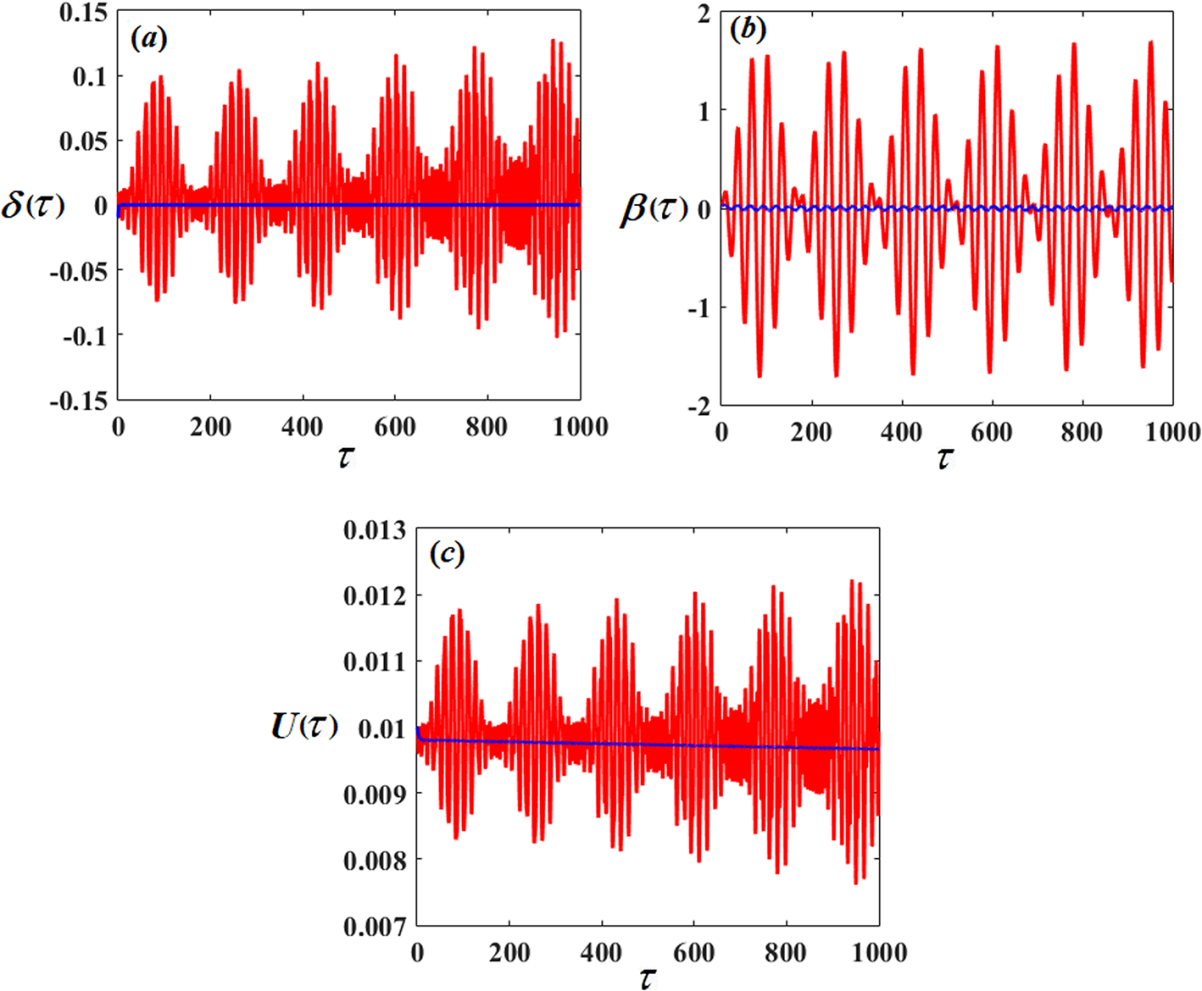
The time response solutions $\delta (\tau ),\,\beta (\tau )$and $U(\tau )$ with resonance cases ${p_1} \approx 2,\,\,{p_2} \approx \omega$ with and without control.

**Fig. 8:**
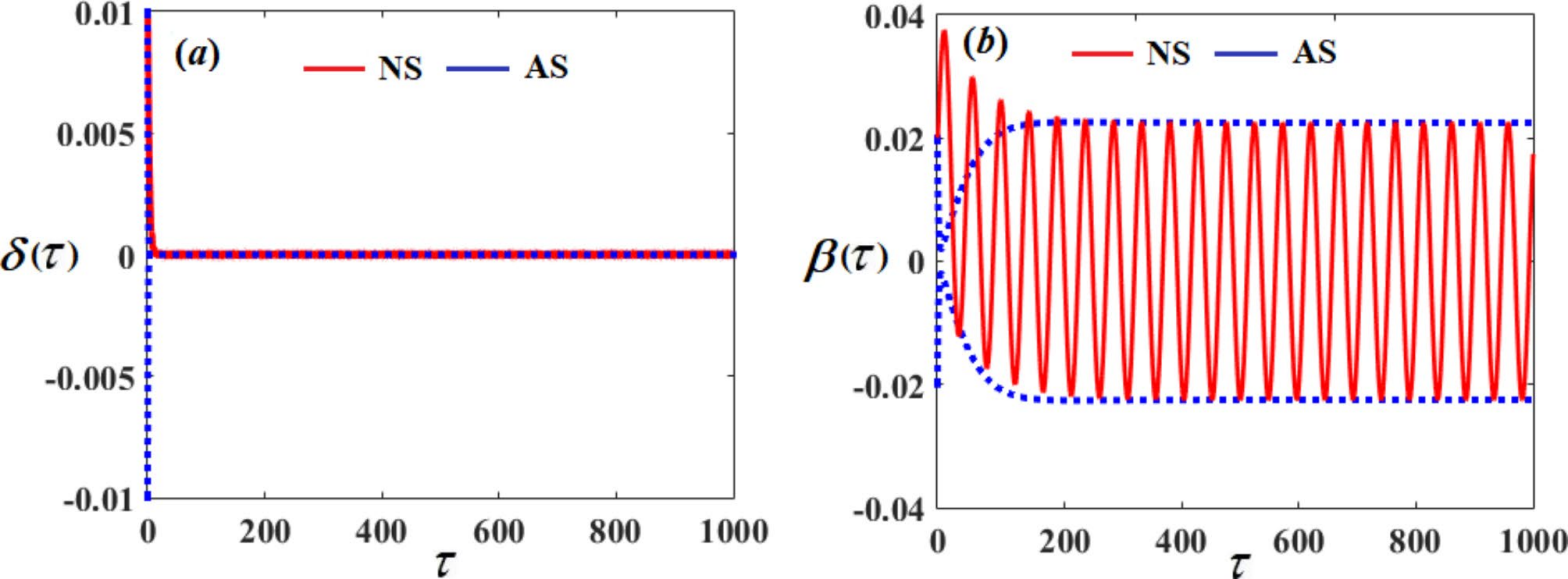
The comparison between NS and AS.

**Fig. 9:**
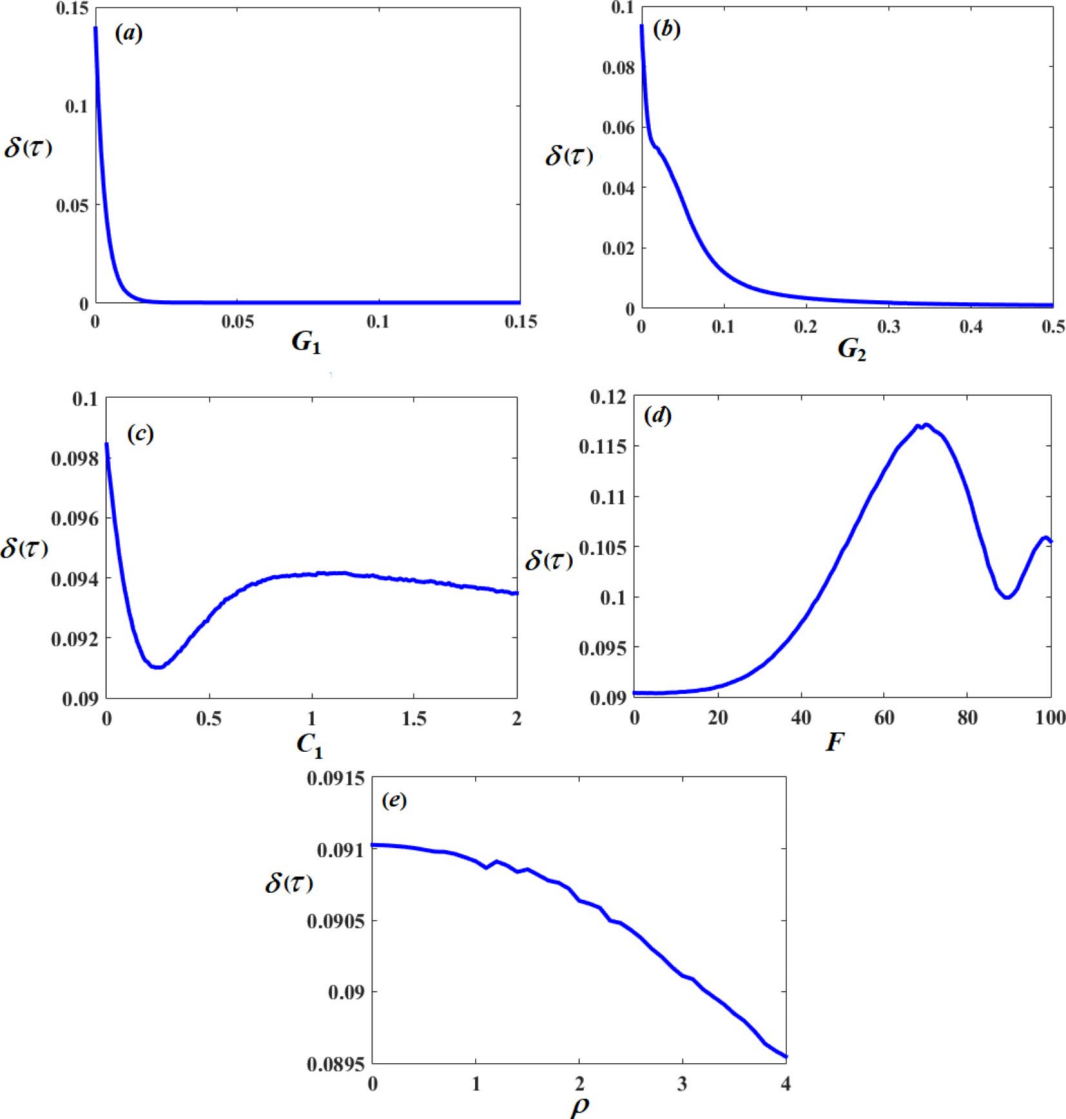
Effect of system parameters ${G_1},{G_2},{C_1},F,$ and $\rho$ on system amplitude $\delta (\tau ).$

**Fig. 10:**
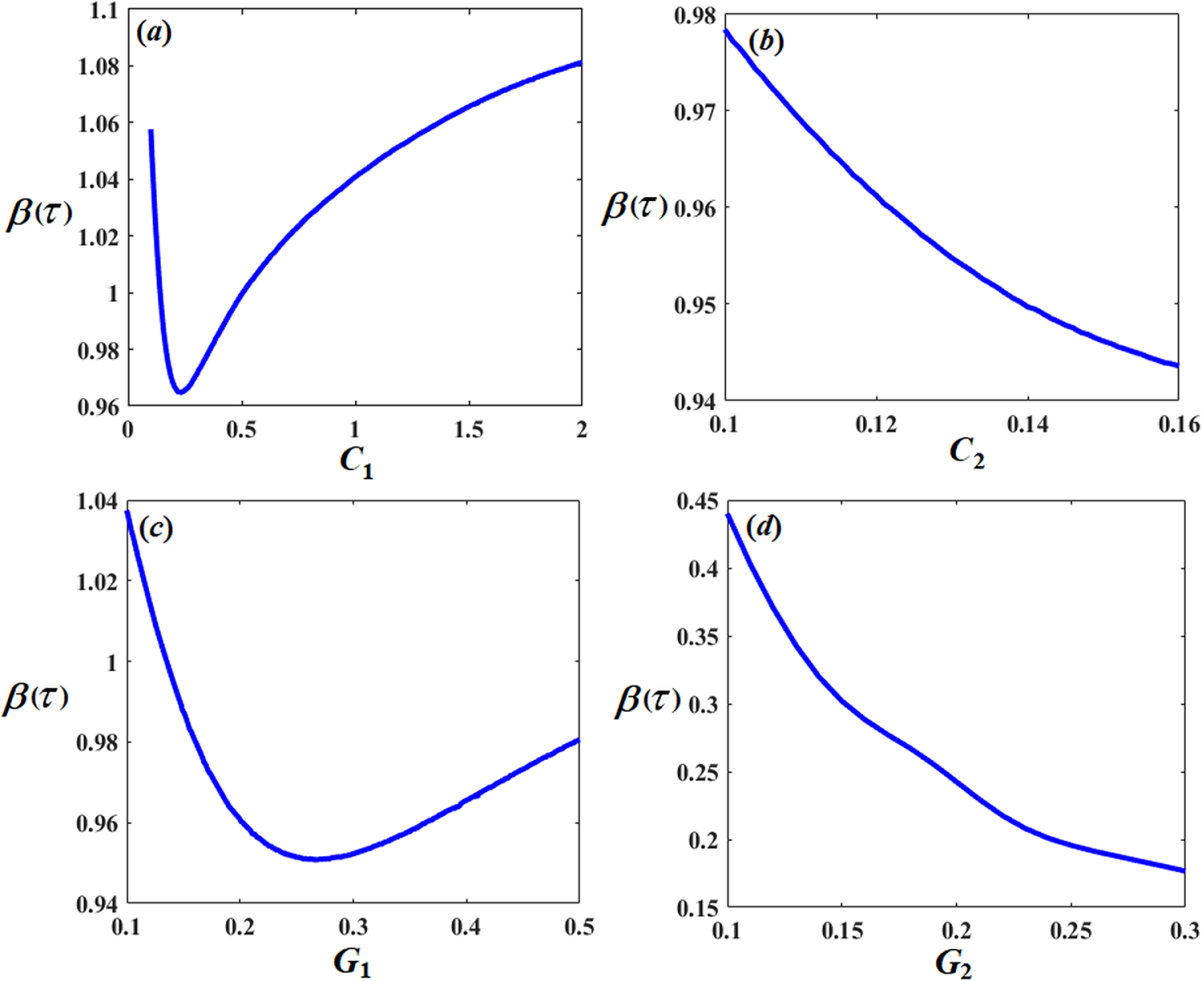
Effect of system parameters ${C_1},{C_2},{G_1},$ and ${G_2}$ on system amplitude $\beta (\tau ).$

Therefore, Figs. 3, 4, 5, 6, 7, 8, 9 and 10 are plotted, in which curves in Figs. 3 and 4 show the time history of the represented waves of the functions $(\delta ,\,\beta ,\,U)$ and phase portraits of $(\delta ,\,\beta )$, respectively, at resonance without control $({G_1}=0,\,{G_2}=0)$. We observe that the amplitudes of these waves have the values $0.1064,\,1.632,$and $0.01201$, respectively, which indicates a threat to the system, hence the importance of using control to limit and reduce these harmful vibrations to maintain the system.

On the other hand, Figs. 5 and 6 represent the time histories and phase portraits of the same functions, at resonance with the NVF controller$({G_1}=3,\,{G_2}=2)$. Notably, the values of the mentioned magnitudes are decreased to $9.567*{10^{ - 7}},\,0.02259,$ and $0.009721$. To clarify this essentially, a comparison was made with and without control, as shown in Fig. 7. We found that the amplitude of $\delta (\tau )$ reduced by $99.999\,\%$, while the amplitude of $\beta (\tau )$ reduced by $98.615\,\%$ and the amplitude of $U(\tau )$reduced by $10\,\%$ due to the effect of the NVF controller represented by $( - {G_1}\,\dot {\chi })$ and $( - {G_2}\,\dot {\beta })$. It is noted that the effect of control is powerful on vibrations$\delta (\tau )$and $\beta (\tau )$, while it is small in voltage $U(\tau )$. This is desirable because the main purpose of control is to reduce dynamical vibrations so that we can benefit from the resulting voltage. The deduction from the parts of Fig. 8 shows a great degree of consistency between AS and NS while also confirming the great accuracy of the MSS.

Let us show the change in amplitudes of $\delta (\tau )$ and $\beta (\tau )$ with varying of the system parameters ${G_1},\,{G_2},\,{C_1},{C_2},\,F,$ and $\rho$. It is noted that the value of the amplitude $\delta (\tau )$ decreases with the increase of ${G_1},\,{G_2},\,{C_1},$ and $\rho$ values. Whereas, it increases with the increase of *F* until a certain value, then the system collapses after that with raising it, as shown in Fig. 9. Furthermore, the amplitude of $\beta (\tau )$ decreases with increase of $\,{C_1},{C_2},\,{G_1},\,$and ${G_2}$ as shown in Fig. 10.

## Steady-state strategies

This section’s primary focus is on the solutions for the steady-state condition of the dynamical system under study. In this case, the solutions would appear at the end of the transient processes. In the current instance, we consider the zero value of $\frac{{d{q_a}}}{{d\tau }}=\frac{{d{\eta _a}}}{{d\tau }}=0\,\,\,\,(a=1,2)$^42^. Thus, the following set of algebraic equations can be created by using the system of equations (40)

41 $$\begin{gathered} 4{q_1}({\sigma _1} - {H_9})+{H_2}{q_1}q_{2}^{2}+2{f_1}{q_1}\cos {\eta _1}=0, \hfill \\ 2{q_1}({c_1}\,+{G_1}+{H_{10}}) - {f_1}{q_1}\sin {\eta _1}=0, \hfill \\ 8{q_2}{\sigma _2}+\frac{1}{\omega }{q_2}({H_3}q_{2}^{2}+{H_2}q_{1}^{2})+\frac{4}{\omega }{f_2}\cos {\eta _2}=0, \hfill \\ {q_2}({c_2}+{G_2}) - \frac{1}{\omega }{f_2}\sin {\eta _2}=0. \hfill \\ \end{gathered}$$

According to an analysis of the last system (41), upon removing the modified phases ${\eta _a}$ from this system, yields the below equations of frequency responses

42 $$\begin{aligned} f_{1}^{2} & =4{({c_1}\,+{G_1}+{H_{10}})^2}+4{({\sigma _1} - {H_9}+\frac{1}{4}{H_2}q_{2}^{2})^2}, \\ f_{2}^{2} & ={\omega ^2}q_{2}^{2}{({c_2}+{G_2})^2}+\frac{1}{4}{(8\omega {q_2}{\sigma _2}+{H_3}q_{2}^{3}+{H_2}q_{1}^{2}{q_2})^2}. \\ \end{aligned}$$

**Fig. 11:**
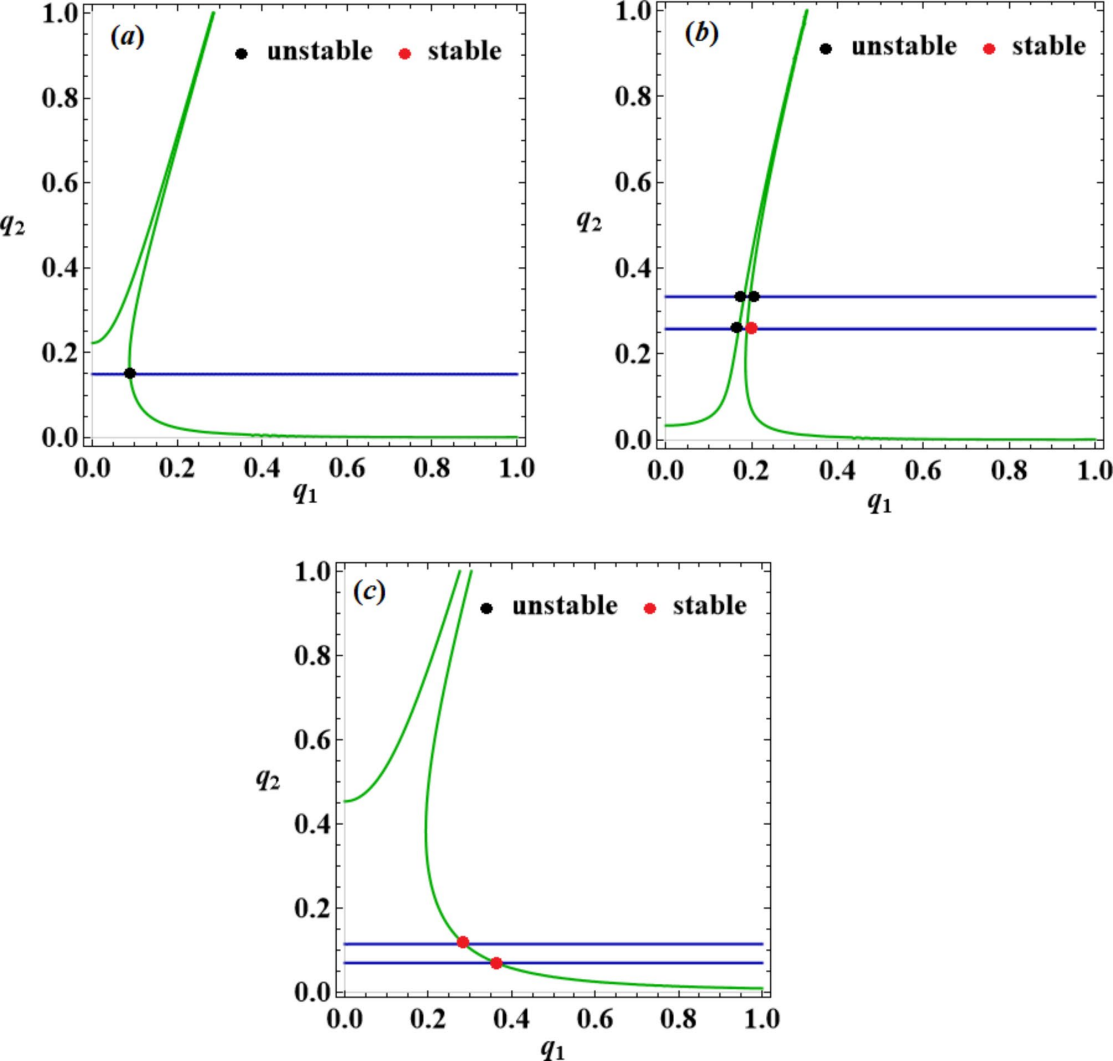
The steady-state case solutions, where they are computed to disclose: (**a**) one fixed point when ${\sigma _1}=0,{\sigma _2}=0$, (**b**) four fixed points when ${\sigma _1}=0.009,{\sigma _2}=0.01$ and (**c**) two fixed points when${\sigma _1}=0.001,\,{\sigma _2}=0.001$.

Notably, every possible steady-state solution might be described for the amplitudes ${q_a}$ at ${\sigma _1}={p_1} - 2$ and ${\sigma _2}={p_2} - \omega$. The roots of the system’s first and second equations (42) are portrayed by the green and blue curves, respectively, as shown in Fig. 11. As stated otherwise, these curves present the locations of the equations’ roots, and the places at which the two curves intersect are the fixed points that correspond to the solutions at the steady-state scenario. These points provide an unquestionable formula for estimating the axial amplitudes and managing vibrations in the steady-state. Moreover, oscillations in a steady state may be unstable or stable. Red points are assigned to indicate the unstable fixed points, while green points are utilized to indicate the stable fixed points, as shown in Table 1.

**Table 1:** Demonstrates the positions of the fixed points of the roots of the system’s first and second equations (42).

Figure	Fixed point	Stable/Unstable	${\sigma _a}\,\,(a=1,2)$
Figure 11a	(0.087,0.1528)	Unstable	${\sigma _1}=0,\,\,{\sigma _2}=0$
Figure 11b	(0.178,0.33)	Unstable	${\sigma _1}=0.009,\,\,{\sigma _2}=0.01$
	(0.197,0.33)	Unstable	
	(0.1667,0.26)	Unstable	
	(0.19,0.26)	Stable	
Figure 11c	(0.2814,0.1167)	Stable	${\sigma _1}=0.001,\,\,{\sigma _2}=0.001$
	(0.3616,0.07318)	Stable	

For a deeper understanding of the system’s stability, we will analyze its behavior near the fixed points by examining minor changes in amplitude and phase.

43 $$\begin{gathered} {q_1}={q_{10}}+{q_{11}},\,\,\,\,\,\,\,\,{q_2}={q_{20}}+{q_{21}},\,\,\, \hfill \\ {\eta _1}={\eta _{10}}+{\eta _{11}},\,\,\,\,\,\,\,{\eta _2}={\eta _{20}}+{\eta _{21}}, \hfill \\ \end{gathered}$$

where ${q_{10}},{q_{20}},{\eta _{10}},$ and ${\eta _{20}}$ represent the solutions in a steady state, whereas ${q_{11}},{q_{21}},{\eta _{11}}$ and ${\eta _{21}}$ indicate the small perturbations compared to ${q_{10}},{q_{20}},{\eta _{10}}$ and ${\eta _{20}}$. After linearization, we can get the following ODE system by substituting (43) into (40)

44 $$\begin{aligned} {q_{10}}\frac{{d{\eta _{11}}}}{{d\tau }} & ={q_{11}}({\sigma _1} - {H_9})+\frac{{{H_2}}}{4}{q_{11}}q_{{20}}^{2}+\frac{{{H_2}}}{2}{q_{10}}{q_{20}}{q_{21}} - \frac{1}{2}{f_1}{q_{10}}{\eta _{11}}\sin {\eta _{10}}+\frac{1}{2}{f_1}{q_{11}}\cos {\eta _{10}}, \\ \frac{{d{q_{11}}}}{{d\tau }} & = - \frac{1}{2}{q_{11}}({c_1}\,+{G_1}+{H_{10}})+\frac{1}{4}{f_1}{q_{10}}{\eta _{11}}\cos {\eta _{10}}+\frac{1}{4}{f_1}{q_{11}}\sin {\eta _{10}}, \\ {q_{20}}\frac{{d{\eta _{21}}}}{{d\tau }} & ={q_{21}}{\sigma _2}+\frac{1}{{8\omega }}{q_{21}}(3{H_3}q_{{20}}^{2}+{H_2}q_{{10}}^{2})+\frac{{{H_2}}}{{4\omega }}{q_{10}}{q_{20}}{q_{11}} - \frac{1}{{2\omega }}{f_2}{\eta _{21}}\sin {\eta _{20}}, \\ \frac{{d{q_{21}}}}{{d\tau }} & = - \frac{1}{2}{q_{21}}({c_2}+{G_2})+\frac{1}{{2\omega }}{f_2}{\eta _{21}}\cos {\eta _{20}}. \\ \end{aligned}$$

The solutions of this system can be expressed as a linear combination of $\,{\kappa _b}\,{e^{\lambda \tau }}$ where ${\kappa _b}\,\,(b=1,2,3,4)$ representing constants and $\lambda$ being the eigenvalue associated with the perturbation, respectively. It was based on the small perturbation functions ${q_{11}},{q_{21}},{\eta _{11}}$ and ${\eta _{21}}$. Considering the above, when the real parts for the roots of the characteristic equation are negative, the fixed points in (44) are asymptotically stable.

45 $${\lambda ^4}+{\Gamma _1}{\lambda ^3}+{\Gamma _2}{\lambda ^2}+{\Gamma _3}\lambda +{\Gamma _4}=0,$$

where ${\Gamma _b}$ are stated in Appendix (II).

The upcoming conditions must be satisfied for fixed points to be stable, according to the Routh-Hurwitz criteria (RHC)^42^:

46 $$\begin{gathered} {\Gamma _{\text{1}}}>0,\,\,\,\,\,\,\,\,\,\,\,\,\,\,\,\,\,\,\,\,\,\,\,\,\,\,\,\,{\Gamma _{\text{3}}}({\Gamma _{\text{1}}}{\Gamma _{\text{2}}} - {\Gamma _{\text{3}}}) - {\Gamma _{\text{4}}}{\Gamma _{\text{1}}}^{{\text{2}}}>0, \hfill \\ {\Gamma _{\text{1}}}{\Gamma _{\text{2}}} - {\Gamma _{\text{3}}}>0,\,\,\,\,\,\,\,\,\,\,\,\,\,{\Gamma _{\text{4}}}>0. \hfill \\ \end{gathered}$$

It must be mentioned that the RHC is considered a fundamental tool in control theory and stability analysis of linear time-invariant systems. They provide a systematic way to determine the stability of a polynomial equation’s roots without explicitly calculating them. The primary importance of these criteria is the determination of the system’s stability. Furthermore, instead of solving for the roots of the characteristic polynomial (which can be algebraically intensive), they allow for a quicker and more straightforward stability determination by constructing and analyzing the Routh array. Moreover, the mentioned method can find the challenging and complicated roots of higher-order systems by simply breaking down the problem into manageable steps.

## Analyzing stability

**Fig. 12:**
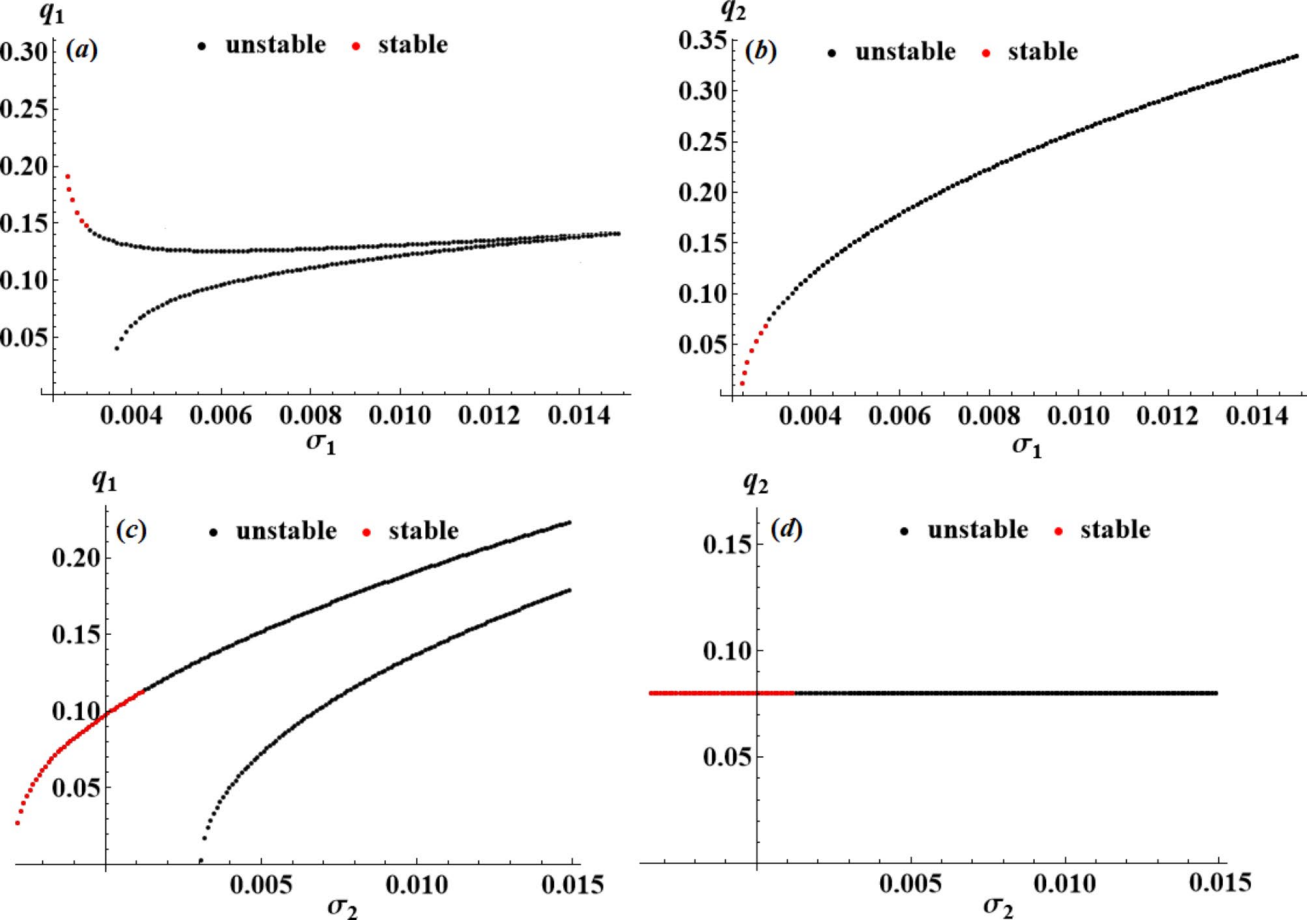
Amplitudes’ resonance curves ${q_a};\,\,(a=1,2)$: (**a**),(**b**) versus ${\sigma _1}\,;\,{\sigma _2}=0.003$, (**c**),(**d**) versus ${\sigma _2}\,;\,{\sigma _1}=0.003$ at ${G_1}=0.0002,\,{G_2}=0.0001.$

**Fig. 13:**
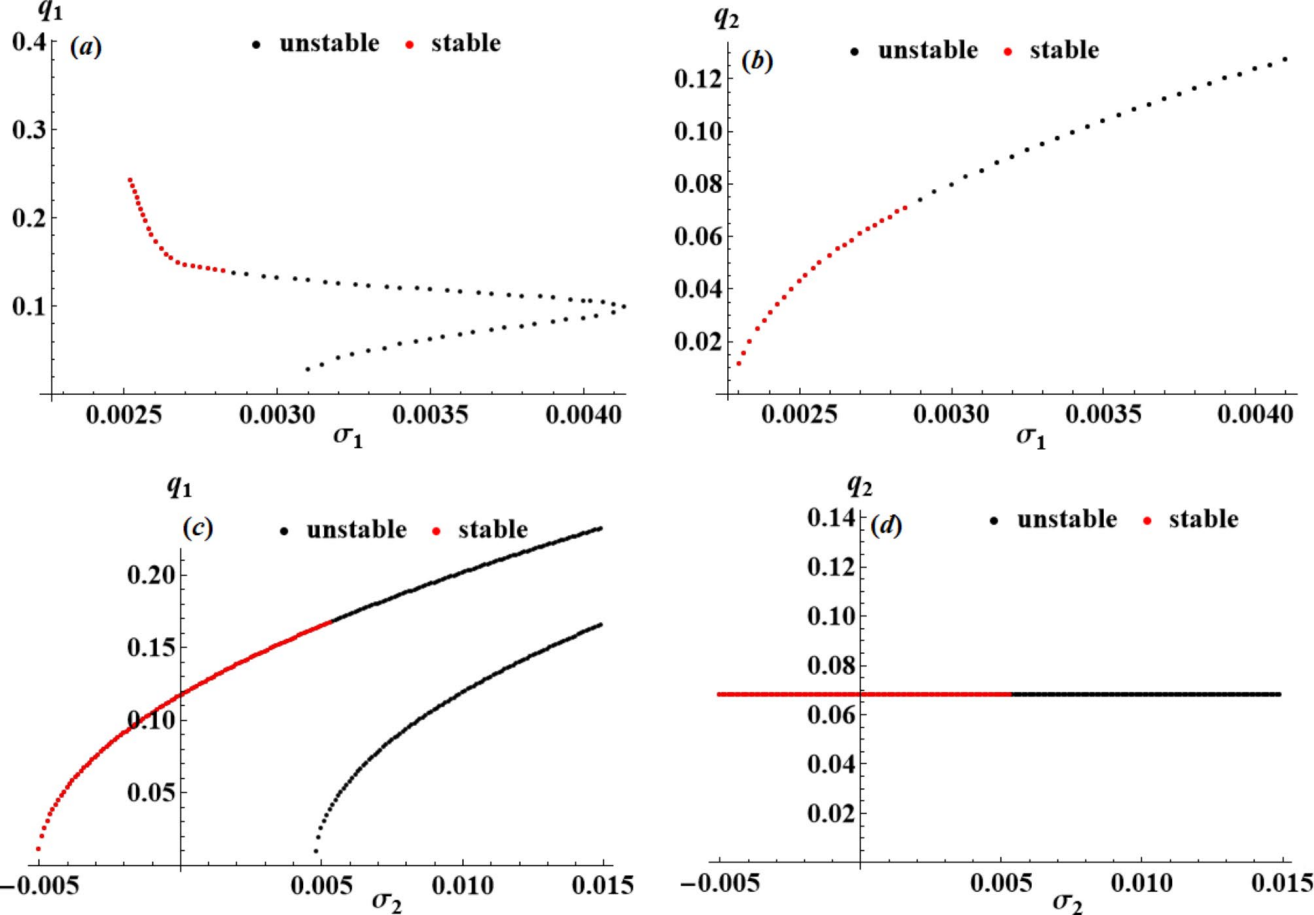
Amplitudes’ resonance curves ${q_a};\,\,(a=1,2)$: (**a**),(**b**) versus ${\sigma _1}\,;\,{\sigma _2}=0.003$, (**c**),(**d**) versus ${\sigma _2}\,;\,{\sigma _1}=0.003$ at ${G_1}=0.0009,\,{G_2}=0.0001.$

**Fig. 14:**
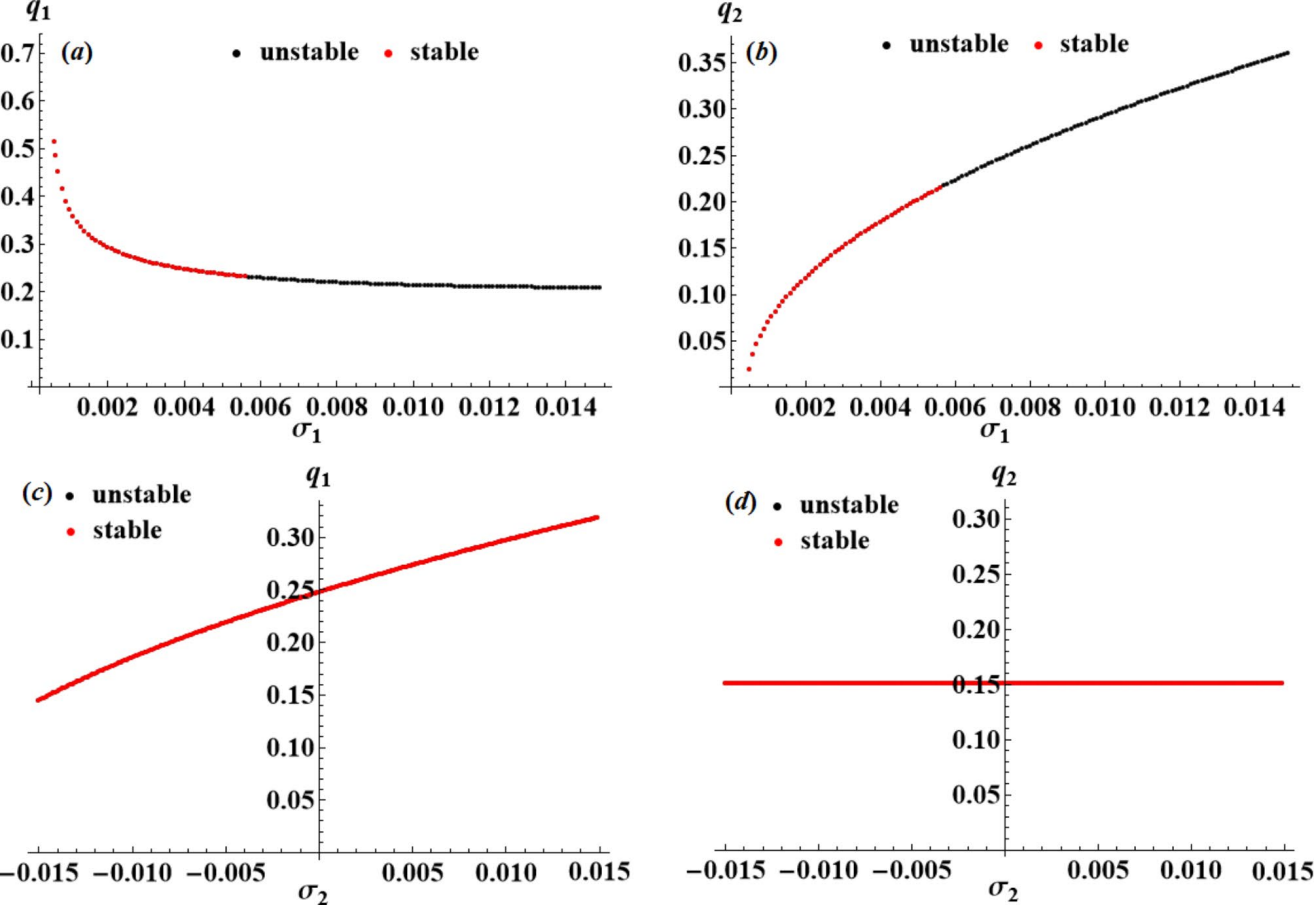
Amplitudes’ resonance curves ${q_a};\,\,(a=1,2)$: (**a**),(**b**) versus ${\sigma _1}\,;\,{\sigma _2}=0.003$, (**c**),(**d**) versus ${\sigma _2}\,;\,{\sigma _1}=0.003$ at ${G_1}=0.0001,\,{G_2}=0.001.$

The purpose of this section is to use the nonlinear stability approach^43^ to evaluate the nonlinear stability of the analyzed model. Using resonance response curves generated from the NS of (42), we will show the stability regions of the fixed points, which are presented in Figs. 12, 13 and 14. The following parameters were used for developing these curves.

$$\omega =0.22,\,{f_1}=0.005,\,{f_2}=0.0001,\,\,{c_1}=0.0001,\,\,{c_2}=0.0002,\,{p_1}=2,\,{p_2}=\omega .$$

It is important to remember that black points denote unstable fixed points, whereas red points signify stable ones. These figures conclude that as the control value increases, the stability zone increases, confirming the influential role the utilized control plays.

## Bifurcation analysis and Lyapunov exponent spectrums

Bifurcation curves are graphical representations of the behavior of a dynamical system as a parameter changes. They indicate points where qualitative changes occur in the system’s behavior, like the creation of novel points of equilibrium or periodic orbits. On the contrary, Lyapunov analysis evaluates the dynamical model’s stability by examining the behavior of solutions in its vicinity. Lyapunov functions are used to determine whether small perturbations from an equilibrium point lead to convergence or divergence over time. Together, bifurcation curves and Lyapunov analysis provide insight into the behavior and stability of dynamical systems, helping to predict and understand complex behaviors such as chaos or stability transitions^44^. The bifurcation diagrams of $\delta (\tau )$, $\beta (\tau )$, and Lyapunov exponent spectrums (LEs) of ${\lambda _b}\,\,(b=1,2,3,4)$ with the amplitudes of excitations ${f_1}$ and ${f_2}$ have been performed to show the many types of motion.

**Fig. 15:**
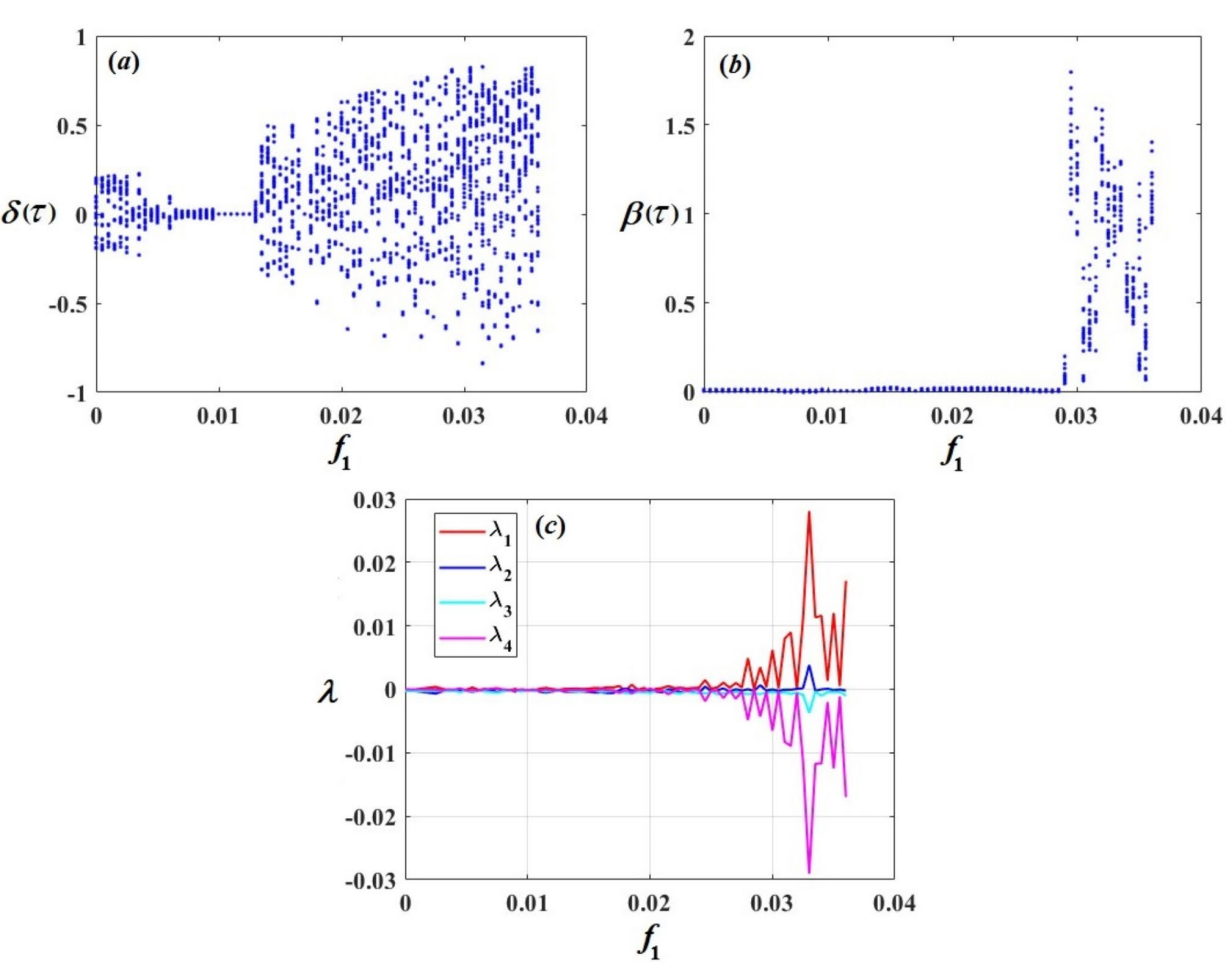
$\delta (\tau )$ and $\beta (\tau )$’s bifurcation diagrams and their LE spectrum with the amplitude of excitation ${f_1}$.

**Fig. 16:**
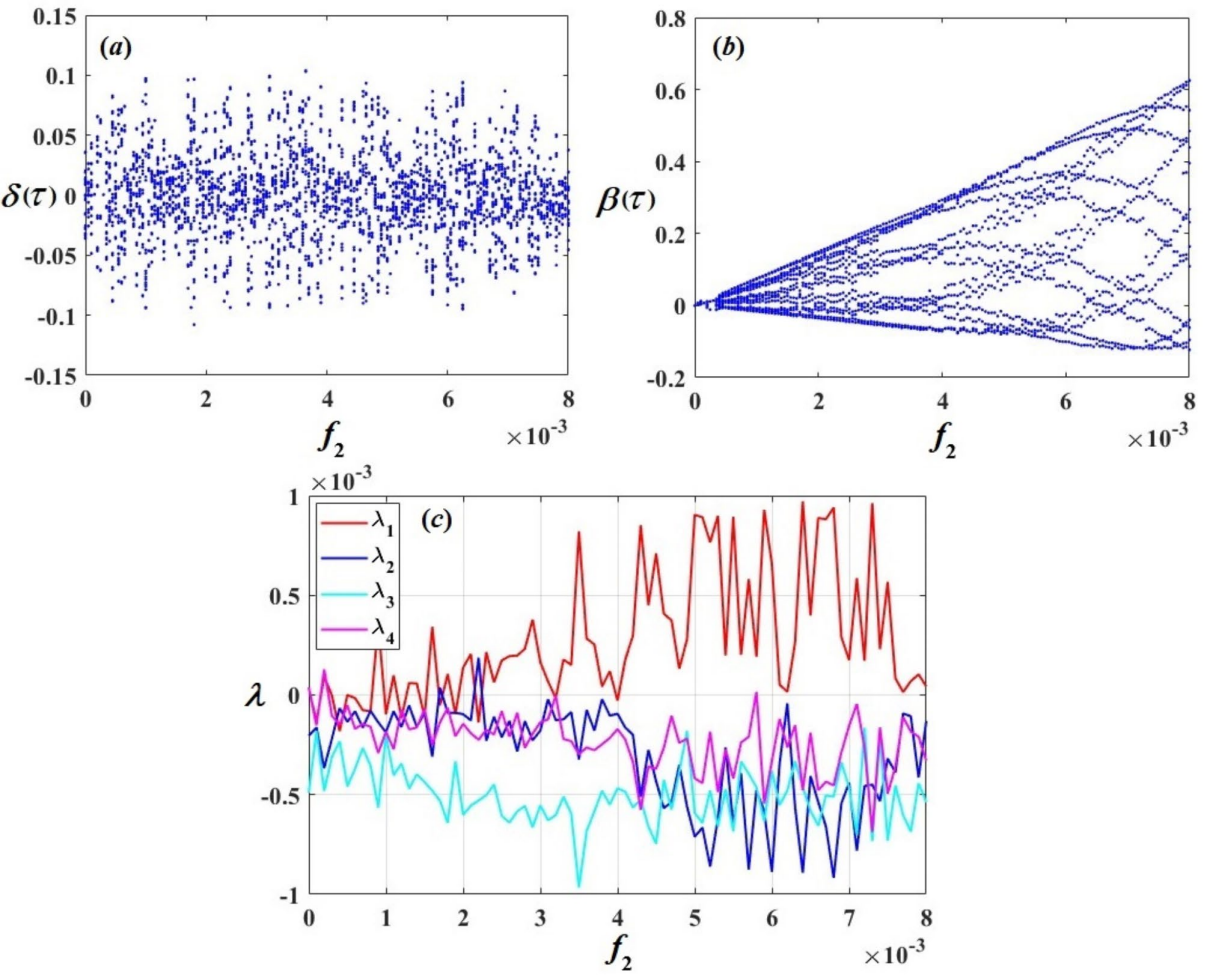
$\delta (\tau )$ and $\beta (\tau )$’s bifurcation diagrams and their LE spectrum with the amplitude of excitation ${f_2}$.

The bifurcation diagrams of $\delta (\tau )$ and $\beta (\tau )$ with the amplitude of excitation ${f_1}$ are presented in Fig. 15a,b, and c shows the LEs spectra that characterize several kinds of motion. According to the diagrams in Fig. 15, we have periods of the excitation amplitude ${f_1}$, every period points to various types of motion of the system. The range at ${f_1} \in [0,0.0065)$ the spectrum of LEs in this range of values is about under the axis $\lambda =0$ or equal to zero, implying that the motion follows a quasi-periodic pattern. This observation is supported by the diagram of bifurcation of $\delta (\tau )$ and $\beta (\tau )$ for the same range of ${f_1}$. Notably, in Fig. 15b, the system appears almost like a straight line. During the periods at ${f_1} \in [0.0065,0.008]$ and $(0.011,0.035]$, we observed that the spectrum of LEs has positive and negative values. Consequently, the system is in chaotic motion, as simultaneously shown in the diagrams of bifurcation for $\delta (\tau )$ and $\beta (\tau )$ especially in Fig. 15a. But the period at ${f_1} \in (0.008,0.011]$ the spectrum of LEs in this range of values is about under the axis $\lambda =0$, which suggests that the motion is periodic. As concurrently displayed in the diagrams of bifurcation for $\delta (\tau )$ and $\beta (\tau )$. Figure 16 represents the bifurcation diagrams of $\delta (\tau )$ and $\beta (\tau )$ with the amplitude of excitation ${f_2}$ and the LEs are plotted. It is observed that the motion is periodic at the range of ${f_1} \in [0,0.0002)$ and $(0.0003,0.0008]$ because the spectrum of LEs in this range of values is about under the axis $\lambda =0$ and the bifurcation diagrams confirm this observation, especially in Fig. 16b, but the motion is chaotic at the range of ${f_2} \in [0.0002,0.0003]$ and $(0.0008,0.008]$ due to the fact that the LE spectrum contains both positive and negative values and reinforced by the diagram of bifurcation of $\delta (\tau )$ and $\beta (\tau )$.

**Fig. 17:**
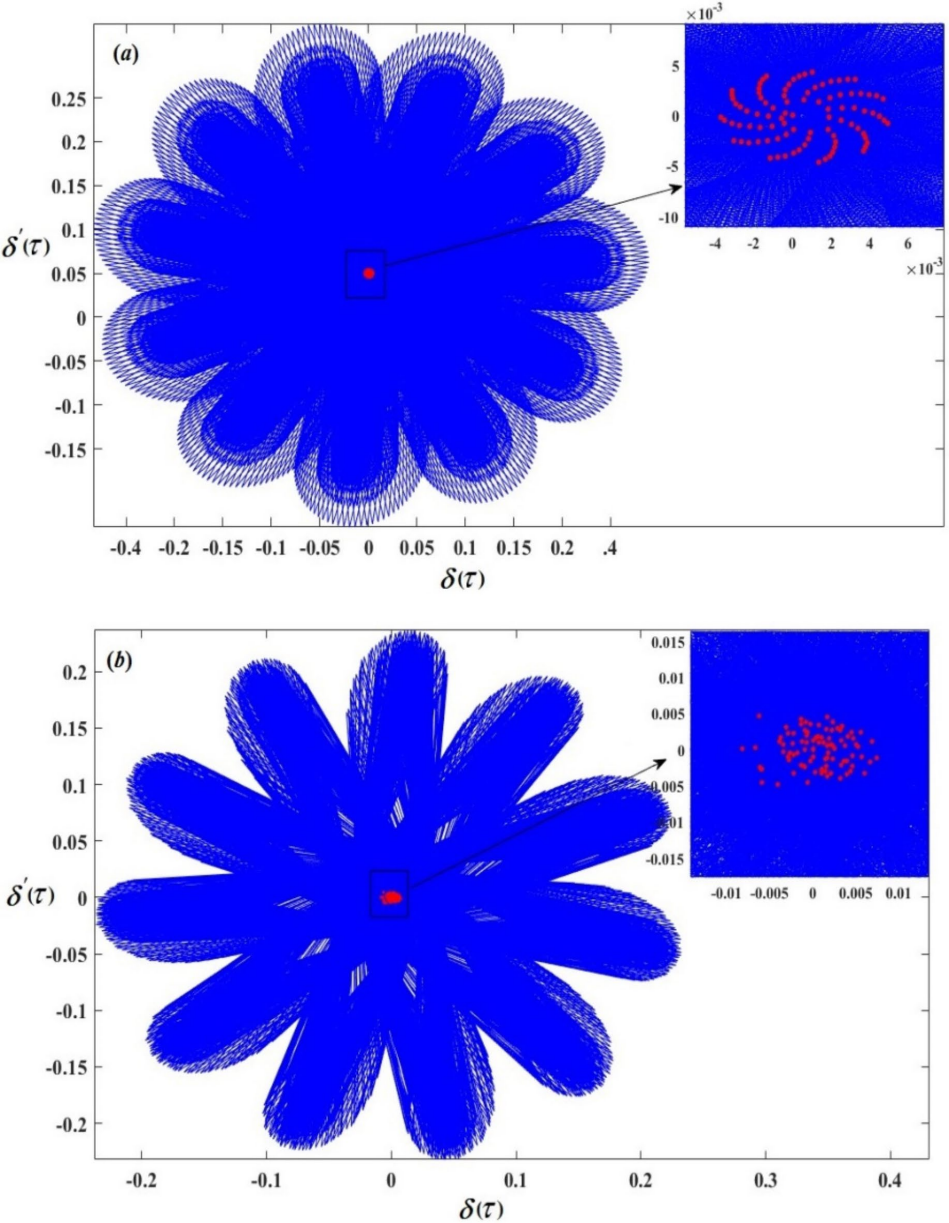
Poincaré maps and phase portraits of $\delta (\tau )$ (**a**) at ${f_2}=0.0005$ and (**b**) at ${f_2}=0.008$.

**Fig. 18:**
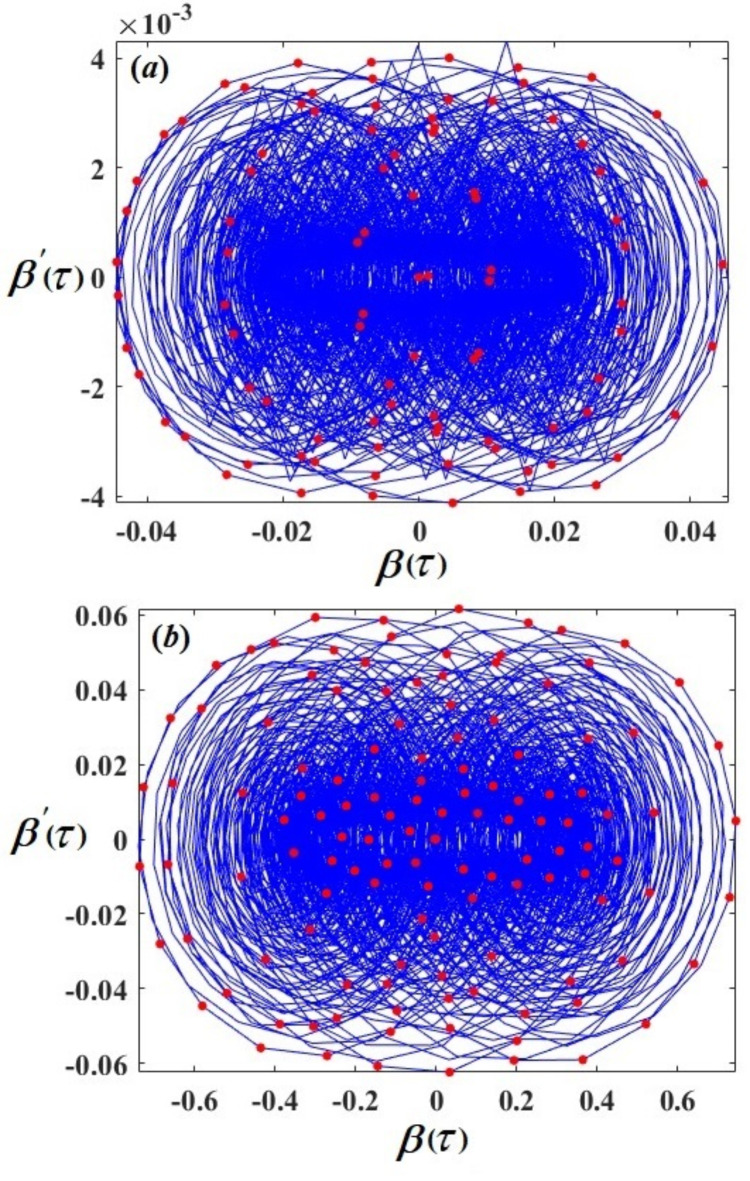
Poincaré maps and phase portraits of $\beta (\tau )$: (**a**) at ${f_2}=0.0005$ and (**b**) at${f_2}=0.008$.

Figures 17 and 18 display the phase portraits and Poincare´ maps; the phase portraits are indicated by blue curves, while the Poincare´ maps are indicated by red dots. To illustrate the different motions of the system, the obtained figures were shown for varying values of the excitation amplitude ${f_2}$. We examine a value ${f_2}=0.0005$ in Figs. 17a and 18a. The red-dot pattern exhibited by Poincaré map closely resembles a closed curve of $\delta (\tau )$ and $\beta (\tau )$, contributing to the earlier conclusion regarding the quasi-periodic motion of the system. The chaotic state that surfaced in the ${f_2}$ values, causes the red dots at ${f_2}=0.008$, as in Figs. 17b and 18b to randomly diverge.

## The piezoelectric device’s performance

This section aims to examine the impact of the piezoelectric device on the 2DOF dynamical system’s behavior and to evaluate its role in electrical energy production. The dielectric materials used in this device are capable of polarization due to mechanical stress generated by the vibrating of the dynamic model. In our scenario, these materials polarize and create a field of electricity. Consequently, this gadget and the dynamical model are used to convert mechanical energy into the necessary electrical energy. The energy-collecting device generates electrical energy that can be utilized for various purposes, including medical remote sensing, emergency medical response monitoring, aerospace applications, military use, and structural monitoring.

**Fig. 19:**
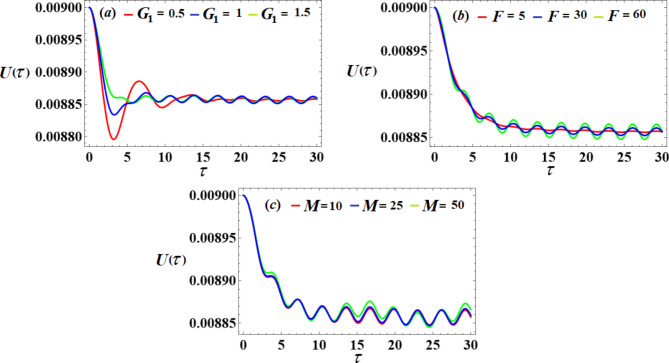
The voltage’s behavior via $\tau$ at the diverse values of ${G_1},F$ and *M*.

**Fig. 20:**
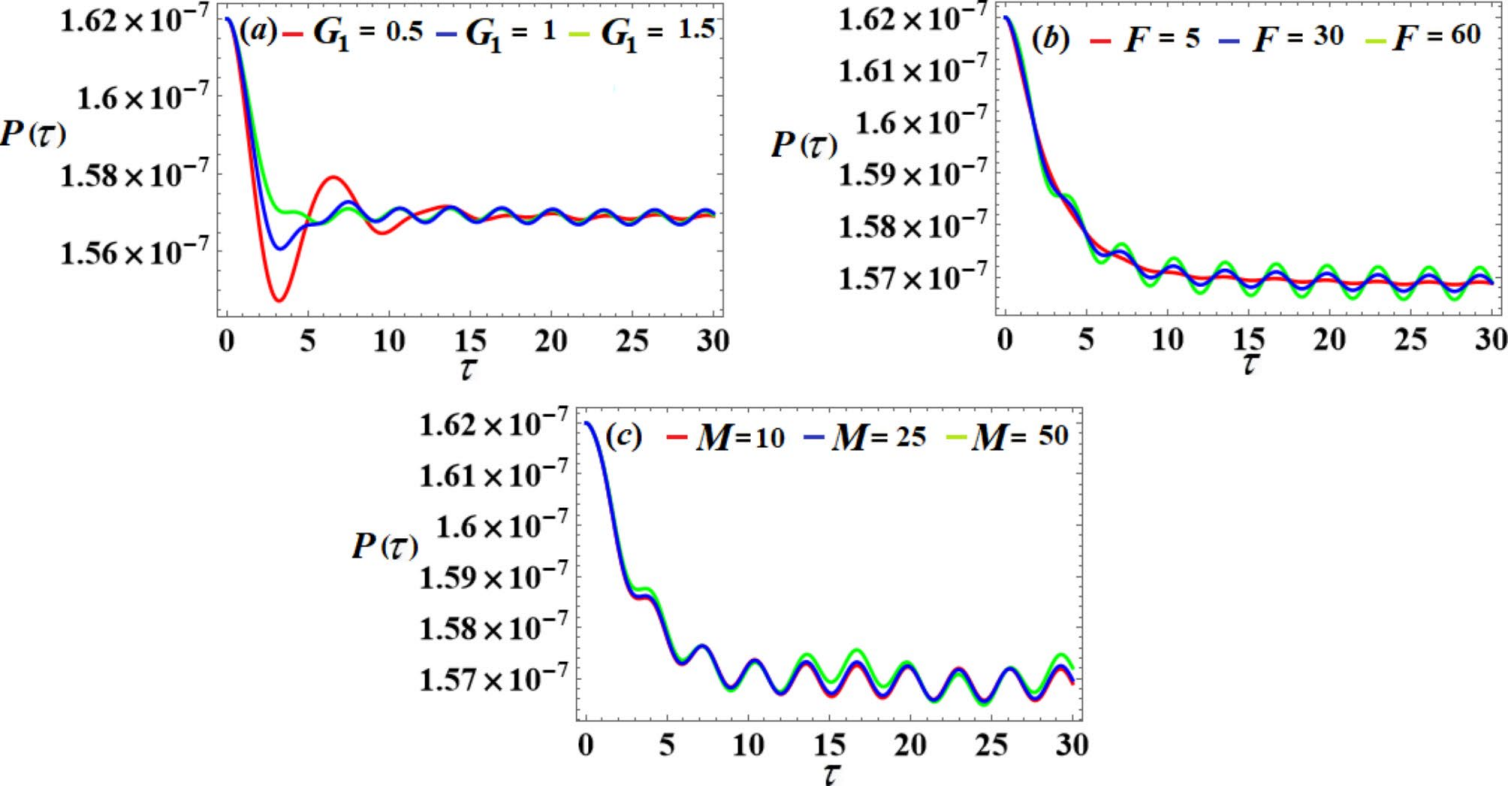
The output power’s behavior via $\tau$ at the different values of ${G_1},F$ and *M*.

**Fig. 21:**
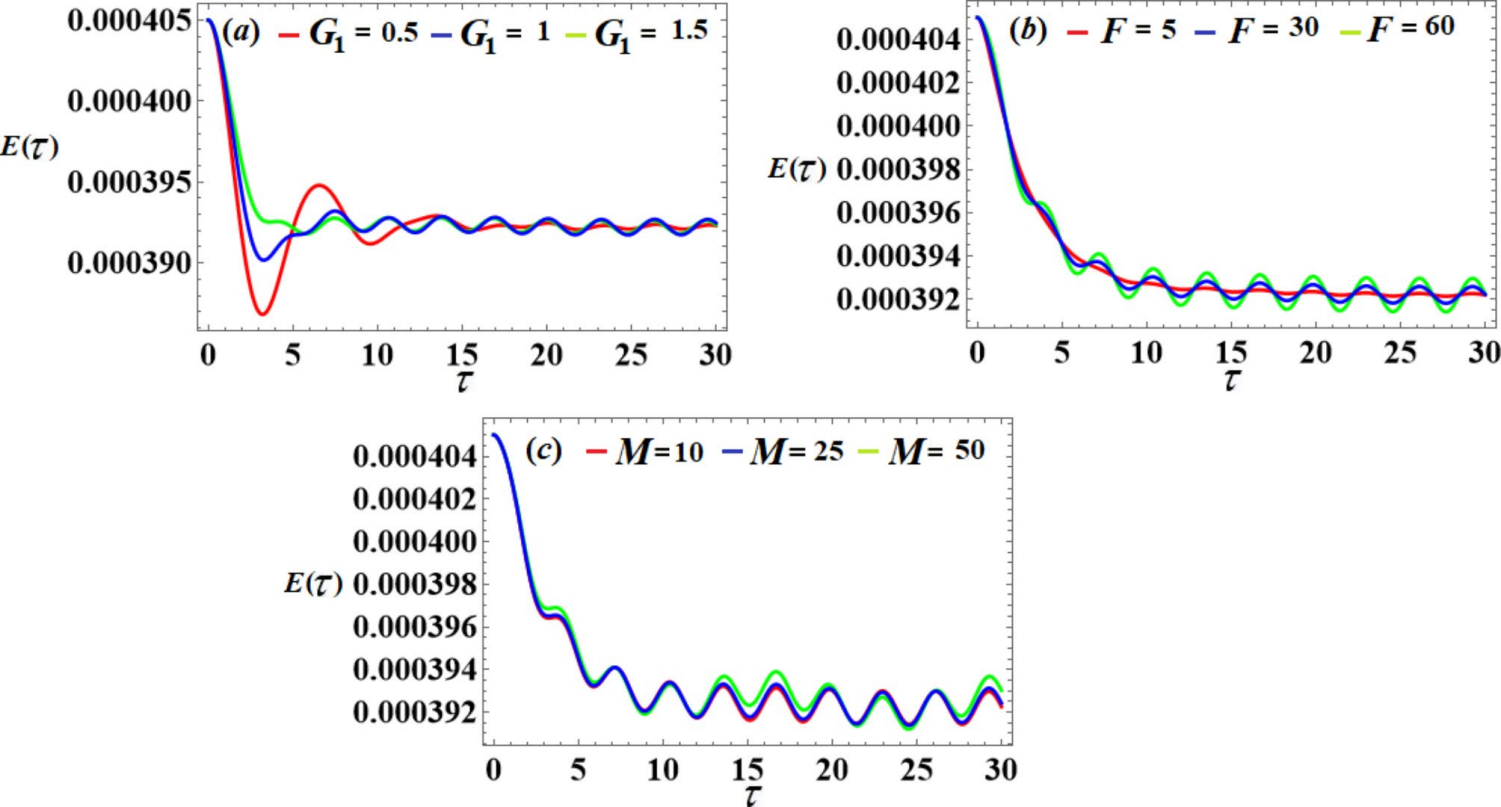
The output energy’s behavior via $\tau$ at the different values of ${G_1},F$ and *M*.

We will examine the effects of various parameter values on the system to achieve optimal performance. The piezoelectric’s voltages, powers, and generated energy over time are shown in Figs. 19 and 20, and 21, respectively. A closer look at individual portions of these figures shows that standing waves have been decaying periodically over the whole duration. As the control gain ${G_1}$ increases, the amplitudes of these waves decrease and increase with the values of *F* and *M* lead to an increase in the amplitudes.

It must be noted that Table 2 provides comparisons between the benchmark work and other prior works, such as^39,40^. These comparisons cover the design, the pivot point’s state, the number of DOF, the derived solutions, stability regions, fixed points, NS, bifurcation diagrams, Lyapunov exponent spectrums, Poincaré maps, and the EH device.

**Table 2:** Comparison between the current work and previous ones in^39,40^.

No.	Comparison items	The current study	The examined work in [39]	The investigated work in [40]
1	DOF	2DOF	3DOF	2DOF
2	Spring’s linearity	Nonlinear	Linear	Linear
3	Pivot point	fixed	in a motion	fixed
4	AS	The AS are obtained	The AS are obtained	The AS are obtained
5	Stability	The stability and instability areas are investigated according to RHC	Not achieved	The stability and instability areas are examined
6	Fixed points	All fixed points are examined	Not achieved	These points are checked
7	NS	The NS are obtained	The NS are achieved	Not achieved
8	Bifurcation diagrams	The bifurcation diagrams have been graphed and discussed	Not achieved	Not achieved
9	Lyapunov exponent spectrums	The Lyapunov exponent spectrums have been achieved and analyzed	Not achieved	Not achieved
10	Poincaré maps	The Poincaré maps are plotted and examined	Not plotted	Not plotted
11	EH device	The influence of a piezoelectric EH device is taken into account.	Not considered	Not considered

## Conclusion

The dynamical system of a 2DOF nonlinear damped harmonic spring pendulum linked with a piezoelectric EH device has been studied. The NVF controller is used to reduce any potentially harmful vibrations. Lagrange’s equations are applied to obtain the governing equations, which are then analytically solved using the MSS until the third-order approximation. These results have graphs to support them and have been verified numerically using the RK-4 technique. ME are obtained, and all acquired external resonance cases are evaluated. Three distinct movements of the system are illustrated and investigated by the bifurcation diagrams, Poincaré maps, and the LEs: quasi-periodic, periodic, and chaotic motion. It is observed that at various values of the excitation amplitude of a parametric external force, gains of the NVF controller, and damping coefficient, we may obtain high output voltage, energy, and power of the piezoelectric device graphically. In addition, the resonance response curves are used to illustrate the system’s stability and identify its stable and unstable fixed points. It was discovered that as a result of the NVF controller represented by$( - {G_1}\,\dot {\chi })$ and $( - {G_2}\,\dot {\beta })$, the amplitude of $\delta (\tau )$and $\beta (\tau )$decreased by $99.999\,\%$ and $98.615\,\%$, respectively, while there was a very slight change in the voltage $U(\tau )$. This is helpful as reducing dynamic vibrations is the main goal of the controller, in addition to benefiting from the resulting voltage from the piezoelectric EH device. The dynamical system under investigation is significant because it can be used for a variety of purposes, such as microphones, sensors in the medical field, actuators in consumer electronics (printers, speakers), and actuators in the industrial sector.

## Electronic supplementary material

Below is the link to the electronic supplementary material.

Supplementary Material 1

## Data Availability

All data generated or analysed during this study are included in this published article.

## Abbreviations and symbols list

DOF: Degree-of-freedom
NVF: Negative-velocity-feedback
EOM: Equations of motion
AS: Approximate solutions
MSS: Multiple-scales-strategy
NS: Numerical solutions
RK-4: The fourth order Runge-Kutta Method
ME: Modulation equations
EH: Energy harvesting
RHC: Routh-Hurwitz criteria
NDF: Negative derivative feedback
PPF: Positive position feedback
VF: Velocity feedback
CVF: Cubic velocity feedback
NCVF: Negative cubic velocity feedback
PD: Proportional derivative
*m*: The attached mass to the spring’s pendulum
$F(t,\,\delta )$: Parametric external force
${M_\beta }(t)$: The acting moment
${k_L}$: Linear stiffness of the spring
${k_{NL}}$: Nonlinear stiffness of the spring
$O\,$: Spring’s suspension point
$(F,\,M)$ and $({\Omega _1},{\Omega _2})$: The magnitudes and frequencies of the applied force and torque
$\delta (t)$: The spring’s elongation
$\beta (t)$: The rotation angle
$${C_1},\,{C_2}$$: Damping coefficients
${l_i}$: Pendulum’s normal length
*g*: The gravity’s acceleration
${R_L}$: The resistive load of the piezoelectric circuit
${c_L}$: The piezoelectric capacitance
$\rho$: The linear coupling coefficient for the piezoelectric circuit
${G_1},\,{G_2}$: Gains of the Negative velocity feedback controller
*U*: The voltage of the load${R_L}$
$\,V,\,T$: Potential and kinetic energies
${\delta _s}$: The spring’s static elongation
$$\varepsilon$$: Small parameter
*P*: The output power of the piezoelectric circuit
*E*: The output energy of the piezoelectric circuit
