# Supplementary material for: Stability and bifurcation analysis of a 2DOF dynamical system with piezoelectric device and feedback control
Source: Sci Rep. 2024 Nov 2;14:26477. doi: 10.1038/s41598-024-75342-z (PMC11531538; doi:10.1038/s41598-024-75342-z)
Supplement: Supplementary file 1 — Supplementary Material 1 [file 41598_2024_75342_MOESM1_ESM.pdf]

Appendices

Appendix (I)

$$H_1 = \frac{\tilde{R}_p \omega_1 \, l \, \tilde{\gamma} \, \tilde{\mu}}{(\omega_1 \tilde{c}_p \tilde{R}_p - i)},$$

$$H_2 = \frac{2\,\omega^2 (4 - 7\omega^2)}{(4\omega^2 - 1)},$$

$$H_3 = \frac{\omega^2 (24\omega^4 - 5\omega^2 - 1)}{2(4\omega^2 - 1)},$$

$$H_4 = \frac{\omega^2 (\omega + 2)(3\omega + 2)}{(2\omega + 1)},$$

$$H_5 = \frac{\omega^2 [(8 - 3\omega)\omega - 4]}{(2\omega - 1)},$$

$$H_6 = -\frac{\omega(\omega + 1)(\omega + 2)(\omega + 3)}{(2\omega + 1)},$$

$$H_7 = \frac{\omega(\omega - 1)(\omega - 2)(\omega - 3)}{(2\omega - 1)},$$

$$H_8 = \frac{\omega^2 (67\omega^2 - 1)}{(24\omega^2 - 6)},$$

$$H_9 = \frac{\mu \, l \, \rho \, c_L R_L^2 \omega_1^2}{(1 + \omega_1^2 c_L^2 R_L^2)},$$

$$H_{10} = \frac{\mu \, l \, \rho \, R_L \omega_1}{(1 + \omega_1^2 c_L^2 R_L^2)}.$$

Appendix (II)

$$\Gamma_1 = \frac{1}{2}(c_1 + c_2 + G_1 + G_2 + H_{10} + \frac{f_2 \sin\eta_{20}}{\omega q_{20}} + \frac{1}{2} f_1 \sin\eta_{10}),$$

$$\begin{aligned}\Gamma_2 = \frac{1}{\omega^2 q_{20}} \{ & \omega \sin\eta_{20} f_2 [0.125 f_1 \sin\eta_{10} + 0.25 G_1 + 0.25 G_2 + 0.25 H_{10}] + \omega c_1 [0.25 f_2 \sin\eta_{20} \\ & + \omega(0.25 c_2 + 0.25 f_1 \sin\eta_{10} + 0.25 G_2) q_{20}] + \omega c_2 [0.25 f_2 \sin\eta_{20} + \omega(0.125 f_1 \sin\eta_{10} \\ & + 0.25 G_1 + 0.25 H_{10}) q_{20}] + \omega^2 q_{20} \{-0.125 f_1^2 + G_2 (0.25 G_1 + 0.25 H_{10}) + f_1 [(0.25 G_1 \\ & + 0.125 G_2 + 0.25 H_{10}) \sin\eta_{10} + (0.25 H_9 - 0.0625 H_2 q_{20}^2 - 0.25\sigma_1) \cos\eta_{10}]\} \\ & + (-0.0625 H_2 q_{10}^2 - 0.1875 H_3 q_{20}^2 - 0.5\omega\sigma_2) f_2 \cos\eta_{20} \},\end{aligned}$$

$$\Gamma_3 = \frac{1}{\omega^2 q_{20}}\{\omega f_2 \sin\eta_{20}\{-0.0625 f_1^2 + G_2(0.125G_1 + 0.125H_{10}) + c_2(0.0625 f_1 \sin\eta_{10}$$
$$+0.125G_1 + 0.125H_{10}) + f_1[\sin\eta_{10}(0.125G_1 + 0.0625G_2 + 0.125H_{10}) + \cos\eta_{10}(0.125H_9$$
$$-0.03125H_2 q_{20}^2 - 0.125\sigma_1)]\} + \omega^2 f_1(c_2 + G_2)q_{20}[\sin\eta_{10}(0.125G_1 + 0.125H_{10}) - 0.0625 f_1$$
$$+\cos\eta_{10}(0.125H_9 - 0.03125H_2 q_{20}^2 - 0.125\sigma_1)] + f_2\cos\eta_{20}[(G_1 + H_{10})(-0.03125H_2 q_{10}^2$$
$$-0.09375H_3 q_{20}^2 - 0.25\omega\sigma_2) + f_1\sin\eta_{10}(-0.015625H_2 q_{10}^2 - 0.046875H_3 q_{20}^2 - 0.125\omega\sigma_2)]$$
$$+c_1\{\omega c_2(0.125 f_2\sin\eta_{20} + 0.125\omega f_1 q_{20}\sin\eta_{10}) + \omega f_1\sin\eta_{10}(0.125\sin\eta_{20} f_2 + 0.125\omega G_2 q_{20})$$
$$+f_2[0.125\omega G_2\sin\eta_{20} + \cos\eta_{20}(-0.03125H_2 q_{10}^2 - 0.09375H_3 q_{20}^2 - 0.25\omega\sigma_2)]\}\},$$

$$\Gamma_4 = \frac{1}{\omega^2 q_{20}} f_1 f_2\{\omega\sin\eta_{20}\{c_1\sin\eta_{10}(0.0625c_2 + 0.0625G_2) + (c_2 + G_2)[-0.03125 f_1 + \sin\eta_{10}$$
$$\times(0.0625G_1 + 0.0625H_{10}) + \cos\eta_{10}(0.0625H_9 - 0.015625H_2 q_{20}^2 - 0.0625\sigma_1)]\} + \cos\eta_{20}$$
$$\times\{c_1\sin\eta_{10}(-0.015625H_2 q_{10}^2 - 0.046875H_3 q_{20}^2 - 0.125\omega\sigma_2) + (G_1 + H_{10})\sin\eta_{10}(-0.015625$$
$$\times H_2 q_{10}^2 - 0.046875H_3 q_{20}^2 - 0.125\omega\sigma_2) + f_1(0.0078125H_2 q_{10}^2 + 0.0234375H_3 q_{20}^2 + 0.0625$$
$$\times\omega\sigma_2) + \cos\eta_{10}[H_2 q_{10}^2(-0.015625H_9 - 0.01171875H_2 q_{20}^2 + 0.015625\sigma_1) + H_3 q_{20}^2(-0.046875$$
$$\times H_9 + 0.01171875H_2 q_{20}^2 + 0.046875\sigma_1) + \omega(-0.125H_9 + 0.03125H_2 q_{20}^2 + 0.125\sigma_1)\sigma_2]\}\}.$$
